# The efficacy and safety of pharmacological treatment for major depressive episode with mixed features specifier: a systematic review and meta-analysis

**DOI:** 10.1017/S0033291725101955

**Published:** 2025-10-07

**Authors:** Sirui Gao, Yanjun Chen, Jin Liu, Qianqian Zhang, Xiaotian Zhao, Bangshan Liu, Yan Zhang, LingJiang Li, Gang Wang

**Affiliations:** 1 Department of Psychiatry, National Clinical Research Center for Mental Disorders, and National Center for Mental Disorders, The Second Xiangya Hospital of Central South University, Changsha, Hunan, China; 2 National Clinical Research Center for Mental Disorders & Beijing Key Laboratory of Mental Disorders, Beijing Anding Hospital, Capital Medical University, Beijing, China

**Keywords:** antidepressants, antipsychotics, major depressive episode, meta-analysis, mixed features specifier, mood stabilizers, systematic review

## Abstract

**Background:**

With the increased prevalence of major depressive episodes with mixed features specifier (MDE-MFS), the pharmacological treatment for MDE-MFS has attracted great clinical attention. This study aimed to investigate the efficacy and safety of medication use for MDE-MFS.

**Methods:**

Commonly used databases were searched for the meta-analysis. Primary efficacy outcomes included response rate and the change in the Young Mania Rating Scale scores; the primary safety outcome was the rate of treatment-emergent hypomania/mania. Effects were expressed as relative risk (RR) or standardized mean difference (SMD).

**Results:**

In patients with MDE-MFS, antipsychotics significantly improved depressive (RR = 1.46 [95% CI: 1.31, 1.61]) and manic (SMD = −0.35 [95% CI: −0.53, −0.17]) symptoms without increasing the risk of manic switch (RR = 0.91 [95% CI: 0.53, 1.55]). However, subgroup analysis of bipolar disorder (BD) patients with MDE-MFS indicated that antipsychotics had limited effects on manic symptoms. Mood stabilizers, especially valproate, demonstrated significant effects in BD patients with MDE-MFS by relieving depressive and manic symptoms. For MDE-MFS in patients with major depressive disorder, trazodone has shown potential effectiveness in retrospective studies, while the effectiveness of antidepressants on BD patients with MDE-MFS lacked evidence.

**Conclusions:**

While antipsychotics are first options for MDE-MFS, their effect on manic symptoms in BD patients with MDE-MFS is still unclear. Mood stabilizers may also be considered, and the use of antidepressants remains a topic of controversy. Since our findings are mostly based on post-hoc analyses, the evidence remains preliminary, highlighting the need for further research to produce more conclusive evidence.

## Introduction

According to the Diagnostic and Statistical Manual of Mental Disorders, Fifth Edition (DSM-5), major depressive episodes with mixed features specifier (MDE-MFS) can be diagnosed when the criteria for MDE are met with at least three manic/hypomanic symptoms. The prevalence of MDE-MFS is particularly high among patients with mood disorders, with a prevalence of 8.92–42.3% for patients with major depressive disorder (MDD) (Grover & Adarsh, 2023; Vázquez et al., 2018) and 11.4–68.7% for those with bipolar disorder (BD) (Grover & Adarsh, 2023; Vázquez et al., 2018). Moreover, the overall prevalence of MDE-MFS is higher than that of manic episodes with mixed features, highlighting the need for increased attention to the treatment of MDE-MFS (Tohen et al., 2017; Vázquez et al., 2018). MDE-MFS often has distinct features, such as a higher incidence in females, earlier onset, more severe depressive symptoms, a higher rate of suicide ideation and attempts, increased irritability, poorer response to pharmacological treatment, and poorer prognosis (McIntyre et al., 2015; Miller et al., 2016; Perugi et al., 2015; Shim, Woo, Jun, & Bahk, 2014; Smith et al., 2009; Tohen et al., 2017; Tondo, Vázquez, Pinna, Vaccotto, & Baldessarini, 2018).

Considering the high prevalence and severe clinical conditions of MDE-MFS, the development of effective pharmacological strategies for this disease becomes crucial. Most guidelines recommend atypical antipsychotics as the primary treatment option for MDE-MFS among patients with BD or MDD, with mood stabilizers as a viable alternative (McIntyre, Suppes, Tandon, & Ostacher, 2017; Rosenblat & McIntyre, 2017; Stahl et al., 2017; Yatham et al., 2021); however, the effectiveness and safety profile of antidepressants remains unclear for MDE-MFS. Although current clinical guidelines provide certain recommendations for the pharmacological treatment of MDE-MFS, these recommendations are predominantly based on expert consensus or indirect evidence from studies focusing on either bipolar depression or mixed episodes in BD, rather than on direct, high-quality evidence specifically targeting the MDE-MFS population. Furthermore, most existing studies on pharmacotherapies for MDE-MFS have failed to provide sufficiently robust evidence to develop safe and effective treatment strategies. A previous meta-analysis on the effectiveness of antipsychotics on MDE-MFS in patients with BD showed that antipsychotics could improve both depressive and manic symptoms (Fornaro et al., 2016). However, this analysis did not demonstrate the effectiveness of atypical antipsychotics on MDE-MFS in patients with MDD, the safety profile of antipsychotics for MDE-MFS, and the efficacy and safety of mood stabilizers and antidepressants for MDE-MFS in patients with MDD or BD.

Collectively, substantial knowledge gaps remain in the pharmacological treatment of these patients. Regarding target populations, a systematic treatment strategy is still lacking for MDD patients with MDE-MFS. Regarding the risk–benefit profile, although current evidence has demonstrated the efficacy of antipsychotics in BD patients with MDE-MFS, their safety profiles warrant further investigation (Fornaro et al., 2016). With respect to treatment options, the efficacy and safety of mood stabilizers is still unclear, and the use of antidepressants remains controversial, primarily due to concerns about inducing affective switching or exacerbating symptoms. Therefore, we conducted a comprehensive systematic review and meta-analysis to investigate the efficacy and safety of pharmacological treatments for MDE-MFS.

## Methods

### Search strategy

Our study protocol was registered with PROSPERO number: CRD42023445953. The selection of subjects for our study was based on the Population-Intervention-Comparison-Outcome (PICO) principle: the population consisted of MDD or BD patients with MDE-MFS; the interventions were pharmacological treatments, including antipsychotics, mood stabilizers, and antidepressants; the comparisons were made between the studied treatment and placebo or positive drug control, and the outcomes were efficacy and safety of the studied treatment. A comprehensive literature search was conducted in PubMed, Embase, PsycINFO, and the Cochrane Library, covering the period from the inception of each database to July 1, 2023, with an updated search on June 1, 2025. Details of the search strategy are presented in the supplementary material.

### Study selection

Our search identified an insufficient number of RCTs for a systematic review or meta-analysis. Therefore, according to the PICO framework, the present study included (1) double-blind, randomized placebo-controlled trials (DB-RCTs), (2) post-hoc analyses from DB-RCTs, (3) case series studies, and (4) observational studies (with or without control groups). The studies were screened according to the Preferred Reporting Items for Systematic Reviews and Meta-Analyses guidelines (Page et al., 2021).

Prior to the standardization of the definition of MDE-MFS in the DSM-5, diagnostic criteria varied across studies. However, all included studies were required to meet the core inclusion criteria of selecting patients who (1) met the DSM-IV or DSM-5 criteria for a major depressive episode, (2) presented with concurrent manic symptoms, and (3) did not meet the full diagnostic criteria for a manic episode. We excluded all studies involving patients with a ‘mixed episode’ as defined by DSM-IV.

### Data extraction and risk of bias assessment

The following data were extracted from studies included in our systematic review by two investigators independently: authors, year of publication, study design, the definition of mixed depression, demographic information (gender distribution, age, and sample size), types of drugs, treatment duration, outcome measurements, and results. We also contacted the authors through e-mail for the key missing data, when necessary.

The risk of bias assessments was determined based on the study design. Different assessment tools and detailed criteria for the risk of bias assessments are presented in the supplementary material.

### Data synthesis

A meta-analysis was conducted to make a quantitative analysis. In the meta-analysis, a random-effects model was used when the heterogeneity among studies was significant (I^2^ statistic >50%); otherwise, a fixed-effect model would be used. Binary outcomes were presented using the pooled relative risk (RR), while continuous outcomes were presented using standardized mean differences (SMDs). Additionally, we computed the number needed to treat (NNT) and the number needed to harm (NNH) to assess clinical benefit and risk. The NNT and NNH were derived from the absolute risk increase (ARI) and were presented only when the pooled RR was significant. (Roose, Rutherford, Wall, & Thase, 2016).

The primary efficacy outcome of depressive symptoms was the clinical response rate, which is defined by the majority of studies as a ≥50% reduction in the total score of Montgomery Asberg Depression Rating Scale (MADRS) at the endpoint (Benazzi et al., 2009; McIntyre, Cucchiaro, Pikalov, Kroger, & Loebel, 2015; McIntyre, Suppes, Earley, Patel, & Stahl, 2020; Patkar et al., 2012; Suppes et al., 2016; Tohen, Kanba, McIntyre, Fujikoshi, & Katagiri, 2014). Two studies also took the improvement in manic symptoms into consideration, suggesting that responders must achieve a ≥50% reduction in the total score of the Mania Rating Scale (MRS) (Patkar et al., 2012) or have fewer than two concurrent manic/hypomanic symptoms at the endpoint (Benazzi et al., 2009). In the present study, the primary efficacy outcome of manic symptoms was the change in the Young Mania Rating Scale (YMRS) score from baseline. The primary safety outcome was the rate of treatment-emergent hypomania/mania (TEM). The secondary safety outcome evaluated the incidence of common adverse events (AEs) associated with antipsychotics, including extrapyramidal symptom (EPS)-related AEs (such as akathisia and parkinsonism), arousal-related AEs (such as insomnia, sedation, somnolence, and drowsiness), and other frequently reported AEs (such as headache, nausea, and vomiting).

Heterogeneity was evaluated using the Q statistic (*p* ≤ 0.1) and the *I* ^2^ statistic (JJ Deeks, Higgins, Altman, McKenzie, & Veroniki, 2024). Sensitivity analysis used the leave-one-out method. Publication bias was shown directly by funnel plot and evaluated by Egger’s test using the STATA 16.0 software, with *p* < 0.05 indicating significant publication bias. We used Review Manager 5.3 for outcome analyses and IBM SPSS 25.0 for the analysis of characteristics of the included studies. The significance level was set at *p* < 0.05 (two-tailed).

## Results

### Literature search and study selection

**Figure 1.**
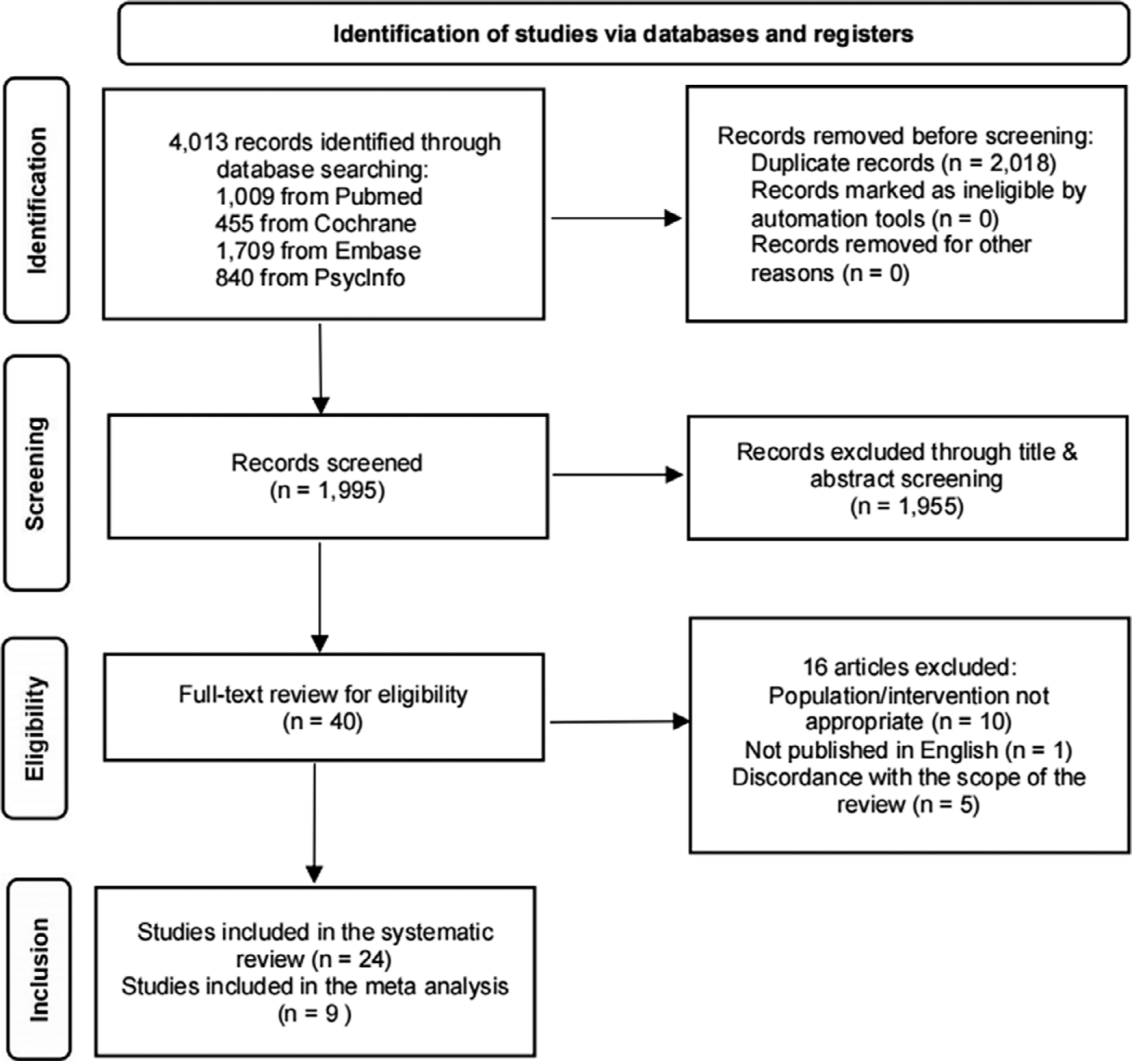
Flowchart of the study selection process.

Out of 4,013 studies screened, 40 were read in full text and 24 were eventually included in this study. The flowchart of the study selection process is shown in Figure 1.

### Study characteristics and quality assessment

Among the 24 included studies, 12 investigated pharmacological treatment for MDE-MFS in MDD, involving a total of 612 patients (mean age: 44.13 ± 13.47 years, percentage of females: 60.1%). Fifteen studies investigated the pharmacological treatment for MDE-MFS in BD, including two DB-RCTs (Durgam et al., 2025; Patkar et al., 2012) and a post-hoc analysis (Pae et al., 2012) that also enrolled both MDD and BD patients with MDE-MFS. The total sample comprised 2,935 BD patients with MDE-MFS, with a mean age of 40.33 ± 11.69 years (excluding one study on adolescents, who were aged 13.6–14.1 years (Singh, Pikalov, Siu, Tocco, & Loebel, 2020)). The characteristics of the included studies are displayed in Supplementary Table 1.

Of the nine studies included in the meta-analysis (Benazzi et al., 2009; Durgam et al., 2025; McIntyre et al., 2020; McIntyre, Cucchiaro, et al., 2015; McIntyre, Durgam, Huo, Kozauer, & Stahl, 2023; Patkar et al., 2012; Singh et al., 2020; Suppes et al., 2016; Tohen et al., 2014), six focused on BD with MDE-MFS (Benazzi et al., 2009; McIntyre et al., 2023, 2020; McIntyre, Cucchiaro, et al., 2015; Singh et al., 2020; Tohen et al., 2014), one focused on MDD with MDE-MFS (Suppes et al., 2016), and two investigated both conditions (Durgam et al., 2025; Patkar et al., 2012). Treatments included olanzapine (Benazzi et al., 2009; Tohen et al., 2014) (*N* = 2), lurasidone (McIntyre, Cucchiaro, et al., 2015; Singh et al., 2020; Suppes et al., 2016) (*N* = 3), cariprazine (McIntyre et al., 2020) (*N* = 1), ziprasidone (Patkar et al., 2012) (*N* = 1), and lumateperone (Durgam et al., 2025; McIntyre et al., 2023) (*N* = 2).

Among the 24 included studies, four were DB-RCTs, one was an open-label study, 11 were post hoc analyses, seven were retrospective studies, and one was a case series study. The overall risk of bias for three DB-RCTs was rated as low, while that for the remaining study was rated as high. Most non-RCTs were of moderate quality. The detailed quality assessment results are summarized in Supplementary Tables 2–6.

### Antipsychotics

#### Overall efficacy and safety for MDE-MFS

**Figure 2.**
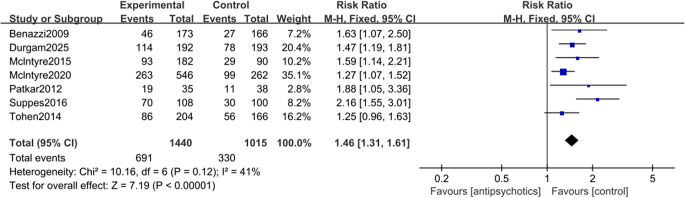
Forest plot for the clinical response rate of antipsychotics versus placebo in the treatment of major depressive episodes with mixed features specifier (MDE-MFS).

**Figure 3.**
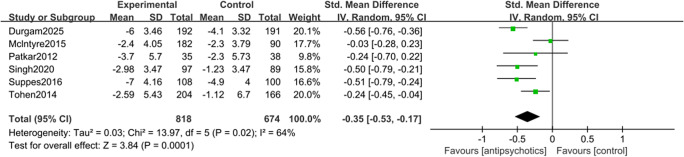
Forest plot for changes in the Young Mania Rating Scale (YMRS) score from baseline in patients with major depressive episodes with mixed features specifier (MDE-MFS) treated with antipsychotics versus placebo.

Seven studies contributed to the meta-analysis of clinical response rate (Benazzi et al., 2009; Durgam et al., 2025; McIntyre et al., 2020; McIntyre, Cucchiaro, et al., 2015; Patkar et al., 2012; Suppes et al., 2016; Tohen et al., 2014). Overall, the result showed that antipsychotics outperformed placebo with regard to clinical response rate (RR = 1.46 [95% CI: 1.31, 1.61], *p* < 0.001; NNT = 7 [95% CI: 5, 9]; Figure 2), and the robustness of the result had been validated through sensitivity analysis. The Egger’s test (*p* = 0.108) and funnel plot (Supplementary Figure 1) indicated that there was no publication bias. Data from six studies were available for the other analysis of primary efficacy outcomes. The result showed that the use of antipsychotics was associated with a significant reduction in the YMRS score, as compared to placebo (SMD = −0.35 [95% CI: −0.53, −0.17], *p* < 0.001; Figure 3), and the result was shown stable in the sensitivity analysis. For this analysis, the Egger’s test (*p* = 0.864, Supplementary Figure 2) also indicated no publication bias.

No significant heterogeneity was detected in the clinical response rate (*I* ^2^ = 41%, *p* = 0.12), while significant heterogeneity was observed regarding the change in YMRS scores (*I* ^2^ = 64%, *p* = 0.02). To account for potential heterogeneity stemming from the inclusion of both RCTs and post-hoc analyses, subgroup analyses were performed. For clinical response, antipsychotic treatment was associated with significantly higher response rates in both RCTs (RR = 1.69, *p* < 0.001) and post-hoc analyses (RR = 1.35, *p* < 0.001; Supplementary Figure 3). For manic symptoms, RCTs demonstrated a significant improvement (SMD = −0.51, *p* < 0.001), whereas post-hoc analyses failed to achieve statistical significance (SMD = −0.25, *p* = 0.05; Supplementary Figure 4).

Subgroup analyses by study design revealed persistent heterogeneity in post-hoc analyses of YMRS score changes (*I^2^* value = 66%, *p* = 0.05). After exclusion of the study on adolescents by Singh et al. (2020), the heterogeneity became statistically non-significant (*I^2^* value = 19%, *p* = 0.29) without significant change in the results (subgroup SMD = −0.21, *p* = 0.15; pooled SMD = −0.27, *p* = 0.007; Supplementary Figure 5). These findings suggested that this particular study might represent a potential source of heterogeneity.

**Figure 4.**
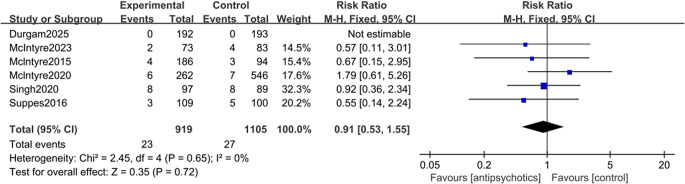
Forest plot for the rate of treatment-emergent hypomania/mania associated with antipsychotics versus placebo in the treatment of major depressive episodes with mixed features specifier (MDE-MFS).

Six studies were included in the primary safety outcome analysis (Durgam et al., 2025; McIntyre et al., 2023, 2020; McIntyre, Cucchiaro, et al., 2015; Singh et al., 2020; Suppes et al., 2016). The overall TEM rate was not significantly different between the two groups (RR = 0.91 [95% CI: 0.53, 1.55], *p* = 0.65; Figure 4). No significant heterogeneity and publication bias were found among the studies (*I^2^* value = 0%, *p* = 0.68; Egger’s test, *p* = 0.251; Supplementary Figure 6). The result was robust, as confirmed by the sensitivity analysis. Subgroup analyses showed no increased risk of TEM in either RCTs or post-hoc analyses (Supplementary Figure 7). However, antipsychotic treatment was associated with a significantly increased overall risk of common AEs (RR = 2.33 [95% CI: 1.63,3.34], *p* < 0.001; NNH = 17 [95% CI: 11,25]; Supplementary Figure 8); this increased risk was particularly evident for arousal-related AEs (RR = 2.77, *p* = 0.005) and other common AEs (RR = 2.03, *p* = 0.003), with a suggestive trend for EPS-related AEs (RR = 2.56, *p* = 0.06).

#### Efficacy and safety for MDE-MFS in patients with BD

A meta-analysis was conducted to investigate the effectiveness of antipsychotics for MDE-MFS in patients with BD (Benazzi et al., 2009; Durgam et al., 2025; McIntyre et al., 2023, 2020; McIntyre, Cucchiaro, et al., 2015; Singh et al., 2020; Tohen et al., 2014). The results indicated that antipsychotics significantly improved clinical response (RR = 1.35 [95% CI: 1.20, 1.52], *p* < 0.001; NNT = 9 [95% CI: 6, 14], Supplementary Figure 9) and manic symptoms (SMD = −0.29 [95% CI: −0.49, −0.09], *p* = 0.005, Supplementary Figure 10).

Subgroup analyses showed a trend toward efficacy for the RCT (lumateperone) on response rate (RR = 1.32, *p* = 0.06), while post-hoc analyses demonstrated significant benefit (RR = 1.35, *p* < 0.001; Supplementary Figure 11). In contrast, the RCT showed a significant improvement in manic symptoms (SMD = −0.44, *p* = 0.002), but this was not significant in post-hoc analyses (SMD = −0.25, *p* = 0.05; Supplementary Figure 12). Additional post-hoc analyses, which were not included in the meta-analysis, also indicated that antipsychotics improved depressive symptoms while showing no effect on manic symptoms (McIntyre et al., 2023, 2020; Patkar et al., 2012).

A recent RCT pilot study by Wang et al. (2023) suggested that quetiapine monotherapy was effective on both depressive and manic symptoms, with a significant reduction in the MADRS and YMRS scores (changes in the MADRS and YMRS scores: −15.51 and −6.97, respectively, both *p* values <0.05).

Five studies examined the safety of antipsychotics among BD patients with MDE-MFS (Durgam et al., 2025; McIntyre et al., 2023, 2020; McIntyre, Cucchiaro, et al., 2015; Singh et al., 2020), which indicated no significant difference in the TEM rate between antipsychotics (including lumateperone, cariprazine, and lurasidone) and placebo (RR = 1.00, *p* = 1.00; Supplementary Figure 13). However, the use of antipsychotics (lurasidone and lumateperone) was associated with a significantly increased risk of overall AEs (RR = 2.26 [95% CI: 1.48,3.43], *p* < 0.05; NNH = 13 [95% CI: 8, 33]; Supplementary Figure 14), including arousal-related AEs (RR = 2.81, *p* = 0.02) and other common AEs (RR = 1.83, p = 0.01), but not EPS-related AEs (RR = 3.61, *p* = 0.11).

#### Efficacy and safety for MDE-MFS in patients with MDD

The systematic literature search identified only three RCTs and one open-label study involving MDD patients with MDE-MFS (Durgam et al., 2025; Han et al., 2019; Patkar et al., 2012; Suppes et al., 2016). Given the limited available evidence, the findings are presented descriptively with no quantitative synthesis undertaken.

Suppes et al. (2016) reported a higher response rate in the lurasidone group than in the placebo group (64.8% versus 30.0%, *p* < 0.001). Lurasidone also demonstrated superior efficacy to placebo in the treatment of manic symptoms (least squares mean [LSM] change in the YMRS score: −7.0 for lurasidone versus −4.9 for placebo, *p* < 0.001). The post-hoc analyses of this study involved a 3-month extension of lurasidone treatment to evaluate the recovery rates (regarding symptoms and functions) (Goldberg et al., 2017), which supported the sustained effectiveness of lurasidone in improving the recovery rate in MDD patients with MDE-MFS. Patkar et al. (2012) reported a higher response rate in the ziprasidone group than in the placebo group (52.9% versus 28.9%, *p* < 0.05) for patients with MDD or BD II who also had MDE-MFS. In a recent 8-week RCT (Durgam et al., 2025), the use of lumateperone was associated with a significantly higher response rate (63.0% versus 39.1%, *p* = 0.0011) and remission rate (40.2% versus 20.7%, *p* = 0.0046), along with greater improvement in manic symptoms (change in the YMRS score: −6.0 versus −4.1, *p* < 0.001) compared to placebo. Han et al. (2019) investigated the efficacy of augmentative aripiprazole in a naturalistic treatment setting, which showed that aripiprazole augmentation therapy was associated with significant reductions in the MADRS and YMRS scores, thereby supporting the effectiveness of augmentative aripiprazole in the treatment of MDD patients with MDE-MFS.

Suppes et al. (2016) reported no significant difference in the TEM rates (2.8% versus 5.0%, *p* > 0.05) between the lurasidone and placebo groups of MDD patients with MDE-MFS. Furthermore, the incidence of EPS-related AEs did not significantly differ between the antipsychotics and placebo groups in both DB-RCTs (Patkar et al., 2012; Suppes et al., 2016). Moreover, lurasidone was not associated with treatment-related sexual dysfunction (Clayton, Tsai, Mao, Pikalov, & Loebel, 2018). Other common AEs reported by 5% or more subjects were nausea and somnolence. Although the incidences of these AEs were slightly higher in the lurasidone group than in the placebo group, the differences were not statistically significant (nausea: 6.4% versus 2.0%, *p* > 0.05; somnolence: 5.5% versus 1.0%, *p* > 0.05) (Suppes et al., 2016). As reported by Durgam et al. (2025), the use of lumateperone did not increase the risk of TEM, suicidal ideation/behavior, EPS-related AEs and other AEs compared to placebo. However, it was found to be associated with a higher incidence of certain common AEs, including somnolence (12.0% vs 1.1%, *p* = 0.02), dizziness (12.0% vs 4.3%, *p* = 0.07), and nausea (10.9% vs 1.1%, *p* = 0.02). In a study by Han et al. (2019), only three patients treated with augmentative aripiprazole experienced AEs, which were increased appetite, somnolence, and headache.

### Mood stabilizers

#### Efficacy and safety for MDE-MFS in patients with BD

Five studies examined the effectiveness and safety of mood stabilizers, including short-term intravenous (IV) and oral valproate and the combination of valproate or lithium with an antipsychotic for BD patients with MFS (Amodeo, Olivola, & Fagiolini, 2017; Buoli et al., 2021; Federico et al., 2019; Santucci, Amodeo, Fagiolini, & Goracci, 2019; Wang et al., 2023). An open-label study comparing short-term augmentative IV valproate and delorazepam revealed that augmentative IV valproate was more effective than IV delorazepam in the treatment of depression and manic symptoms, with a higher response rate in the valproate group than in the delorazepam group (45% versus 12.5%, *p* = 0.01) (Buoli et al., 2021). Amodeo, Olivola, and Soo (2017) conducted a pilot study assessing the efficacy and safety of short-term IV valproate (mean duration: 3.72 days) in BD patients with MFS, which showed that short-term IV valproate effectively reduced both manic and depressive symptoms, according to the Clinical Global Impressions Scale for Bipolar Disorder (CGI-BP). Two retrospective observa tional studies also indicated a good effect of IV valproate in treating such patients, with significant reductions in the MADRS and YMRS scores among those who received IV valproate (Federico et al., 2019; Santucci et al., 2019). In a RCT pilot study by Wang et al. (2023), for patients with poor response to quetiapine monotherapy, both combination therapies with quetiapine + lithium (QTP + L) and quetiapine + valproate (QTP + V) led to a notable reduction in the MADRS score (QTP + L: −7.18 [95% CI: −13.04, −1.32], *p* = 0.025; QTP + V: -8.6 [95% CI: −15.75, −1.45], *p* = 0.0257), while QTP + L was also associated with a significant reduction in the YMRS score (QTP + L: −4.82, [95% CI: −9.33, −0.31], *p* = 0.047).

Regarding the safety of IV valproate, seven participants (14.0%) experienced mild AEs, including sedation, nausea, dizziness, and dry mouth, as reported by Amodeo et al. (Amodeo et al., 2017 Severe AEs leading to discontinuation of treatment included neutropenia (3.4% (Santucci et al., 2019 and 6.0% (Amodeo et al., 2017), prolonged INR (0.8% (Santucci et al., 2019), and severe tiredness (0.8% (Santucci et al., 2019). No TEM was reported in the above studies.

#### Efficacy and safety for MDE-MFS in patients with MDD

Only one study evaluated the efficacy and safety of adjuvant valproate therapy in MDD patients with MDE-MFS (Liu, 2014). Among the 22 patients included in this study, 18 had significant improvement in depressive symptoms after receiving valproate at an average dose of 462.5 ± 280.5 mg/day. Over a follow-up period from 3 to 60 months, 12 patients reported AEs, among whom four discontinued their treatment due to intolerable AEs. Common AEs reported in this study included dry mouth, headache, flattened emotion, joint pain, hair loss, bitter taste, skin rash, dizziness, and mild elevation of liver enzymes.

### Antidepressants

#### Efficacy and safety for MDE-MFS in patients with BD

Two studies examined the use of adjunctive antidepressants in the treatment of BD patients with MDE-MFS. One study involved a post-hoc analysis comparing the olanzapine/fluoxetine combination (OFC) and the olanzapine monotherapy, which showed a trend toward a higher response rate in patients receiving OFC, although the difference was not statistically significant (OR = 2.00 [95% CI: 0.96, 4.19], *p* > 0.05) (Benazzi et al., 2009). The other study, which utilized data from the Systematic Treatment Enhancement Program for Bipolar Disorder (STEP-BD) (Goldberg et al., 2007), found no significant difference in the time to recovery between patients receiving a mood stabilizer monotherapy and those receiving a mood stabilizer and an antidepressant, suggesting that the augmentation with antidepressants might not provide additional benefits regarding symptom improvement.

A study by Benazzi et al. (2009) found that the switch rate was low and comparable between patients receiving OFC and those receiving olanzapine monotherapy (8.5% versus 6.8%, *p* > 0.05), indicating that adjunctive fluoxetine did not pose a higher risk of TEM. Conversely, Goldberg et al. found that adjunctive antidepressant therapy was associated with more severe manic symptoms through a 3-month follow-up, as compared to monotherapy with a mood stabilizer (Goldberg et al., 2007).

#### Efficacy and safety for MDE-MFS in patients with MDD

Two retrospective studies, which had no control group, investigated the efficacy and safety of trazodone in MDD patients with MDE-MFS (Carmellini et al., 2025; Serro et al., 2019). The first study reported a 51.54% response rate after 8.5 ± 4.13 days of intravenous trazodone administration, with significant reductions in agitation and irritability as measured by corresponding items in YMRS and MADRS. The second study showed significant decreases in both YMRS and MADRS scores (both *p* < 0.05). Both studies indicated favorable safety profiles, with only mild and transient AEs such as somnolence, fatigue, and constipation.

## Discussion

### Overall findings

**Figure 5.**
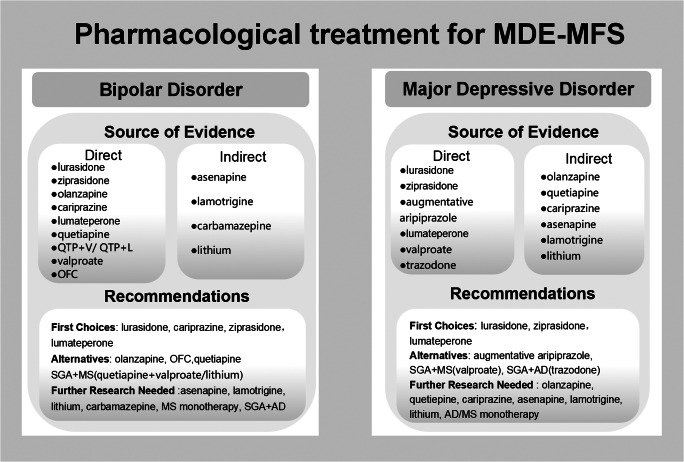
Evidence-based pharmacological treatment for major depressive episodes with mixed features specifier (MDE-MFS) in patients with bipolar disorder (BD) or major depressive disorder (MDD). *Note:* QTP + V, ‘quetiapine plus valproate’; QTP + L, ‘quetiapine plus lithium’; OFC, ‘the olanzapine/fluoxetine combination’; SGA, ‘second-generation antipsychotic’; MS, ‘mood stabilizer’; AD, ‘antidepressant’; BD, ‘bipolar depression’; MDD, ‘major depressive disorder’; MDE-MFS, ‘major depressive episode with mixed features specifier’.

Through the comprehensive analysis, we found that antipsychotics were effective in improving depressive and manic symptoms in patients with MDE-MFS, with a good safety profile and no increased risk of severe AEs. We also found that mood stabilizers and certain antidepressants (trazodone and OFC) appeared to be effective in patients with MDE-MFS, but evidence on their efficacy and safety was limited and controversial due to the small number of studies available. A summary of pharmacological treatments for MDE-MFS, based on both direct and indirect evidence, is presented in Figure 5.

### Treatment strategies for MDE-MFS in patients with BD

Several authoritative guidelines, including the Canadian Network for Mood and Anxiety Treatments and International Society for Bipolar Disorders (CANMAT/ISBD) (Yatham et al., 2021) and the World Federation of Societies of Biological Psychiatry Guidelines for the Biological Treatment of Bipolar Disorders (WFSBP) (Grunze et al., 2018), recommend atypical antipsychotics as the primary treatment for MDE-MFS in patients with BD.

Our meta-analysis demonstrated that antipsychotics significantly improved depressive symptoms in BD patients with MDE-MFS. The findings suggest that antipsychotics, including quetiapine, olanzapine, ziprasidone, lurasidone, cariprazine, and lumateperone, should be considered as first-line treatment options for MDE-MFS. Among them, lurasidone is recommended as the preferred option, a recommendation supported by numerous studies for its efficacy, along with a favorable safety profile characterized by a low risk of manic switch and minimal incidence of serious AEs such as weight gain (Corponi et al., 2019). In addition to its efficacy in adults, lurasidone has demonstrated good effects on both depressive and manic symptoms in children and adolescents with MDE-MFS (Singh et al., 2020) without increasing the risk of TEM. Furthermore, its favorable safety profile in this population has also been confirmed (Solmi et al., 2020), underscoring its therapeutic potential in young people with MDE-MFS. Although olanzapine and quetiapine demonstrated comparable efficacy (Tohen et al., 2014; Wang et al., 2023) and are endorsed by clinical guidelines (Rosenblat & McIntyre, 2017; Stahl et al., 2017; Yatham et al., 2021), they were associated with a higher incidence of AEs such as weight gain and metabolic disorders, as compared to lurasidone (Katagiri et al., 2014; Leucht et al., 2013); these safety concerns render them less preferable options. In addition to guideline-recommended medications, the novel antipsychotic agent lumateperone has demonstrated favorable efficacy and safety in an RCT (Durgam et al., 2025), suggesting its potential qualification as a first-line option. However, its current non-approval status in China may limit its clinical use. Overall, antipsychotics demonstrated favorable safety profiles, with common AEs (e.g. nausea, somnolence) generally being mild and self-limiting.

Notably, our findings revealed inconsistencies in the effects on manic symptoms. The overall analysis showed a significant advantage for antipsychotic treatment (SMD = −0.29, *p* = 0.005); however, subgroup analyses indicated considerable heterogeneity. The single included RCT of lumateperone demonstrated robust efficacy (SMD = −0.44, *p* = 0.002), whereas pooled post-hoc analyses (involving lurasidone, olanzapine, and lumateperone) did not reach significance (p = 0.05). Additionally, three post-hoc analyses evaluating ziprasidone, cariprazine, and lumateperone also demonstrated no significant antimanic effect in this patient population (McIntyre et al., 2023, 2020; Patkar et al., 2012). Differences in study design may account for the discrepancy. Furthermore, other factors also warrant consideration, including variations in pharmacological profiles, the limited number of available studies, the presence of mild manic symptoms, and the unique pathophysiological characteristics of these patients. As MDE-MFS is not simply a co-occurrence of depressive and manic/hypomanic symptoms in patients with BD, treatment must address both symptom domains concurrently. The suboptimal efficacy of certain antipsychotics in managing manic symptoms may contribute to persistence of symptoms, thereby delaying intervention and increasing the risk of treatment resistance. Nonetheless, owing to design limitations, current findings should be interpreted cautiously, and prospective studies are warranted for validation.

While both CANMAT/ISBD and WFSBP guidelines acknowledged the effectiveness of mood stabilizers, they differed in specific recommendations. CANMAT/ISBD recommends the use of divalproex and lamotrigine, whereas WFSBP only recommends carbamazepine. However, there is a lack of evidence supporting the therapeutic effects of lamotrigine and carbamazepine on BD patients with MDE-MFS, and current findings only support the effect of short-term IV valproate/sodium valproate on MDE-MFS in patients with BD. Furthermore, it remains uncertain whether valproate/sodium valproate monotherapy can be regularly used in these patients, as none of the studies specified concomitant use of medications. Evidence also supports the combination therapy with quetiapine and lithium or valproate (Wang et al., 2023); thus, a combination therapy with a mood stabilizer (e.g. valproate or lithium) and an antipsychotic may be beneficial if an antipsychotic monotherapy is found ineffective (Stahl et al., 2017).

Concerning the treatment of MDE-MFS in patients with BD, all guidelines advised that the use of antidepressants alone should be avoided (Grunze et al., 2018; Stahl et al., 2017; Yatham et al., 2021) and that only the OFC is recommended for combination therapy. Despite the conflicting results from the study on the efficacy of the OFC therapy (Benazzi et al., 2009), it should be noted that this does not necessarily mean that combination therapy with antidepressants is suitable for these patients. The positive effects of the OFC therapy may be attributed to its unique mechanism of action in upregulating dopamine and noradrenaline levels in the brain, which makes this combination more effective than monotherapies with drugs contained in this combination (E. D. Deeks & Keating, 2008). The good safety profile of OFC (e.g. it does not increase the risk of TEM) could also be attributed to this unique mechanism, although further research is needed for confirmation. Therefore, it is recommended to avoid using antidepressants alone or in combination in the treatment of MDE-MFS in patients with BD unless clear evidence is provided.

### Treatment strategies for MDE-MFS in patients with MDD

At present, two guidelines have provided pharmacological treatment strategies for MDD patients with MDE-MFS: the Florida Best Practice Psychotherapeutic Medication Guidelines (FPG) (McIntyre et al., 2017) and Guidelines for the Recognition and Management of Mixed Depression (GRMMD) (Stahl et al., 2017). The FPG recommends atypical antipsychotics or mood stabilizers as the initial treatment for MDE-MFS in patients with MDD, while the GRMMD provides more detailed strategies, recommending antipsychotics (e.g. lurasidone, ziprasidone, and aripiprazole) as the first-line treatment and mood stabilizers (e.g. valproate, lamotrigine, and lithium) as the second-line treatment. Notably, the guidelines differ significantly in their recommendations regarding antidepressants. The GRMMD cautions against antidepressant monotherapies for MDE-MFS in patients with MDD, whereas the FPG recommends antidepressant monotherapies with a medication, such as bupropion, vortioxetine, or a selective serotonin reuptake inhibitor as the initial treatment for this patient population.

Antipsychotics such as lurasidone and ziprasidone have demonstrated good efficacy and safety in previous RCTs (Patkar et al., 2012; Suppes et al., 2016), which is further supported by findings in the present study. Furthermore, studies by Goldberg et al. and Pikalov et al. indicated that the severity of manic symptoms at baseline could influence the outcome of patients treated with lurasidone, with those having more severe ‘mixed features’ achieving greater benefits (Goldberg et al., 2020; Pikalov et al., 2017). Similar to most antipsychotics, lurasidone and ziprasidone are also associated with AEs, such as nausea, somnolence, and dizziness, but most of them were mild and tolerable. Augmentative aripiprazole can also be considered, as supported by the study of Han et al. (Han et al., 2019). Similar to its profiles in BD patients with MDE-MFS, lumateperone has exhibited efficacy and safety in MDD patients with MDE-MFS, as demonstrated by an RCT (Durgam et al., 2025), thereby supporting its consideration as a treatment option for this patient population.

There is a notable lack of research on the effectiveness and safety of mood stabilizers in the treatment of MDD patients with MDE-MFS. Through the literature search, we only found a case series study reporting high or moderate improvement in depressive symptoms after treatment with valproate (Liu, 2014). Thus, a comprehensive summary of the use of mood stabilizers for MDE-MFS in patients with MDD remains unavailable.

Only two studies included in the present study investigated the effectiveness of an antidepressant, trazodone, in MDD patients with MDE-MFS (Carmellini et al., 2025; Serro et al., 2019). While the findings indicated that trazodone significantly improved both depressive and manic symptoms of such patients, the conclusion was not compelling enough to be translated into clinical practice due to limitations such as the retrospective design, the use of concomitant medications, and a lack of control groups. The discrepancies between current guidelines remain unresolved based on the available evidence; therefore, further research is necessary to establish the efficacy and safety of antidepressant monotherapies for MDE-MFS in patients with MDD. Nevertheless, the existing evidence does not definitively discourage the use of antidepressants in these patients. At clinicians’ discretion, antidepressants may serve as an adjunctive therapy alongside antipsychotics in the initial treatment, which should also be supplemented by thorough assessments of clinical presentations and potential benefits.

### Advantages and limitations

Compared with previous meta-analyses, this study included more updated studies, which reinforced the findings that antipsychotics are effective in improving depressive symptoms of MDE-MFS in patients with BD and MDD. However, we still found conflicting results regarding the effectiveness of antipsychotics on manic symptoms in BD patients with MDE-MFS. Furthermore, we also assessed the safety of antipsychotics, which may help clinicians make informed decisions by weighing the pros and cons of such treatment for MDE-MFS. This study also delved into the use of mood stabilizers and antidepressants. Since the evidence was derived directly from the population with MDE-MFS, our conclusions may carry greater credibility and clinical relevance.

Some limitations should be acknowledged. First, owing to the relatively small number of pre-specified RCTs, the incorporation of post-hoc analyses in the meta-analysis may affect the overall effect estimates due to potential selection bias and confounding; thus, the findings warrant validation through prospectively designed trials. Second, the operational definitions of ‘mixed features’ varied across studies. Despite this variability, all definitions were grounded in a core principle: the presence of a depressive episode concomitant with subthreshold manic/hypomanic symptoms that did not meet the criteria for a full manic or hypomanic episode. Furthermore, the consistently observed mild manic severity (mean YMRS score < 12) across all studies collectively indicates substantial diagnostic homogeneity. Subgroup analyses further corroborated the robustness of the findings across different diagnostic methods and other conditions (Supplementary Figures 15–17). However, definitional differences may limit the generalizability of the findings to DSM-5-defined MDE-MFS, highlighting the need for standardized diagnostic criteria in future research. Finally, the use of conventional rating scales (MADRS and YMRS), rather than mixed-feature-specific instruments may not fully capture symptom complexity. This limitation highlights the need for specialized assessment tools in future research, including the DSM-5 Clinically Useful Depression Outcome Scale for Mixed Features (Zimmerman, Chelminski, Young, Dalrymple, & Martinez, 2014) and the Koukopoulos Mixed Depression Rating Scale (Sani, Vöhringer, Barroilhet, Koukopoulos, & Ghaemi, 2018).

## Conclusions and prospects

The findings of the present study may complement the existing guidelines. To improve our understanding of the efficacy and safety of pharmacological interventions for patients with MDE-MFS, further DB-RCTs are essential. Further studies on maintenance treatment are also needed to develop a full-course pharmacological treatment strategy for MDE-MFS. Studies on dose and duration optimization are also warranted to refine current pharmacotherapies for MDE-MFS to maximize benefits and minimize risks for patients.

## Supporting information

Gao et al. supplementary material Gao et al. supplementary material

## Supplementary material

The supplementary material for this article can be found at http://doi.org/10.1017/S0033291725101955.
