# Supplementary material for: The efficacy and safety of pharmacological treatment for major depressive episode with mixed features specifier: a systematic review and meta-analysis
Source: Psychol Med. 2025 Oct 7;55:e294. doi: 10.1017/S0033291725101955 (PMC12527531; doi:10.1017/S0033291725101955)
Supplement: Gao et al. supplementary material [file S0033291725101955sup001.docx]

**Supplement**

**The efficacy and safety of pharmacological treatment for major depressive episode with mixed features specifier: a systematic review and meta-analysis**

**Search query: Take PubMed as an example:**

(1)MDE-MFS in BD

(((antipsychotic*[Title/Abstract] OR chlorpromazine[Title/Abstract] OR perphenazine[Title/Abstract] OR haloperidol[Title/Abstract] OR penfluridol[Title/Abstract] OR sulpiride[Title/Abstract] OR perospirone[Title/Abstract] OR lurasidone[Title/Abstract] OR blonanserin[Title/Abstract] OR iloperidone[Title/Abstract] OR Risperidone[Title/Abstract] OR Paliperidone[Title/Abstract] OR Ziprasidone[Title/Abstract] OR clozapine[Title/Abstract] OR Olanzapine[Title/Abstract] OR Quetiapine[Title/Abstract] OR amisulpride[Title/Abstract] OR Aripiprazole[Title/Abstract] OR Asenapine[Title/Abstract]) OR (mood stabilizers[Title/Abstract] OR antimanic*[Title/Abstract] OR lithium carbonate[Title/Abstract] OR valporate[Title/Abstract] OR carbamazepine[Title/Abstract] OR oxcarbazepine[Title/Abstract] OR lamotrigine[Title/Abstract]) OR (antidepressant*[Title/Abstract] OR selective serotonin reuptake inhibitor*[Title/Abstract] OR SSRI[Title/Abstract] OR SSRIs[Title/Abstract] OR fluoxetine[Title/Abstract] OR fluvoxamine[Title/Abstract] OR paroxetine[Title/Abstract] OR sertraline[Title/Abstract] OR citalopram[Title/Abstract] OR escitalopram[Title/Abstract] OR vortioxetine[Title/Abstract] OR serotonin norepinephrine reuptake inhibitor*[Title/Abstract] OR SNRI[Title/Abstract] OR SNRIs[Title/Abstract] OR duloxetine[Title/Abstract] OR venlafaxine[Title/Abstract] OR desvenlafaxine[Title/Abstract] OR milnacipran[Title/Abstract] OR levomilnacipran[Title/Abstract] OR mirtazapine[Title/Abstract] OR mianserin[Title/Abstract] OR nefazodone[Title/Abstract] OR trazodone[Title/Abstract] OR vilazodone[Title/Abstract] OR bupropion[Title/Abstract] OR reboxetine[Title/Abstract] OR agomelatine[Title/Abstract] OR noradrenergic and specific serotonergic antidepressant*[Title/Abstract] OR NaSSA[Title/Abstract] OR NaSSAs[Title/Abstract] OR mirtazapine[Title/Abstract] OR TCA[Title/Abstract] OR TCAs[Title/Abstract] OR tricyclic[Title/Abstract] OR amersergide[Title/Abstract] OR amineptine[Title/Abstract] OR amitriptyline[Title/Abstract] OR amoxapine[Title/Abstract] OR butriptyline[Title/Abstract] OR clomipramine[Title/Abstract] OR clorimipramine[Title/Abstract] OR demexiptiline[Title/Abstract] OR desipramine[Title/Abstract] OR dothiepin[Title/Abstract] OR doxepin[Title/Abstract] OR imipramine[Title/Abstract] OR lofepramine[Title/Abstract] OR melitracen[Title/Abstract] OR metapramine[Title/Abstract] OR nortriptyline[Title/Abstract] OR noxiptiline[Title/Abstract] OR opipramol[Title/Abstract] OR protriptyline[Title/Abstract] OR quinupramine[Title/Abstract] OR tianeptine[Title/Abstract] OR trimipramine[Title/Abstract])) AND (("Bipolar Disorder"[Mesh]) OR ((((((((((((((((((((((((((((Bipolar Disorders[Title/Abstract]) OR (Disorder, Bipolar[Title/Abstract])) OR (Psychosis, Manic-Depressive[Title/Abstract])) OR (Psychosis, Manic Depressive[Title/Abstract])) OR (Psychoses, Manic-Depressive[Title/Abstract])) OR (Psychoses, Manic Depressive[Title/Abstract])) OR (Manic-Depressive Psychosis[Title/Abstract])) OR (Manic Depressive Psychosis[Title/Abstract])) OR (Bipolar Mood Disorder[Title/Abstract])) OR (Bipolar Mood Disorders[Title/Abstract])) OR (Disorder, Bipolar Mood[Title/Abstract])) OR (Mood Disorder, Bipolar[Title/Abstract])) OR (Affective Psychosis, Bipolar[Title/Abstract])) OR (Bipolar Affective Psychosis[Title/Abstract])) OR (Psychoses, Bipolar Affective[Title/Abstract])) OR (Psychosis, Bipolar Affective[Title/Abstract])) OR (Bipolar Disorder Type 1[Title/Abstract])) OR (Type 1 Bipolar Disorder[Title/Abstract])) OR (Manic Depression[Title/Abstract])) OR (Depression, Manic[Title/Abstract])) OR (Depressions, Manic[Title/Abstract])) OR (Depression, Bipolar[Title/Abstract])) OR (Bipolar Depression[Title/Abstract])) OR (Bipolar Disorder Type 2[Title/Abstract])) OR (Type 2 Bipolar Disorder[Title/Abstract])) OR (Manic Disorder[Title/Abstract])) OR (Disorder, Manic[Title/Abstract])) OR (Manic Disorders[Title/Abstract])))) AND (mixed[Title/Abstract])

(2)MDE-MFS in MDD

((antipsychotic*[Title/Abstract] OR chlorpromazine[Title/Abstract] OR perphenazine[Title/Abstract] OR haloperidol[Title/Abstract] OR penfluridol[Title/Abstract] OR sulpiride[Title/Abstract] OR perospirone[Title/Abstract] OR lurasidone[Title/Abstract] OR blonanserin[Title/Abstract] OR iloperidone[Title/Abstract] OR Risperidone[Title/Abstract] OR Paliperidone[Title/Abstract] OR Ziprasidone[Title/Abstract] OR clozapine[Title/Abstract] OR Olanzapine[Title/Abstract] OR Quetiapine[Title/Abstract] OR amisulpride[Title/Abstract] OR Aripiprazole[Title/Abstract] OR Asenapine[Title/Abstract]) OR (mood stabilizers[Title/Abstract] OR antimanic*[Title/Abstract] OR lithium carbonate[Title/Abstract] OR valporate[Title/Abstract] OR carbamazepine[Title/Abstract] OR oxcarbazepine[Title/Abstract] OR lamotrigine[Title/Abstract]) OR (antidepressant*[Title/Abstract] OR selective serotonin reuptake inhibitor*[Title/Abstract] OR SSRI[Title/Abstract] OR SSRIs[Title/Abstract] OR fluoxetine[Title/Abstract] OR fluvoxamine[Title/Abstract] OR paroxetine[Title/Abstract] OR sertraline[Title/Abstract] OR citalopram[Title/Abstract] OR escitalopram[Title/Abstract] OR vortioxetine[Title/Abstract] OR serotonin norepinephrine reuptake inhibitor*[Title/Abstract] OR SNRI[Title/Abstract] OR SNRIs[Title/Abstract] OR duloxetine[Title/Abstract] OR venlafaxine[Title/Abstract] OR desvenlafaxine[Title/Abstract] OR milnacipran[Title/Abstract] OR levomilnacipran[Title/Abstract] OR mirtazapine[Title/Abstract] OR mianserin[Title/Abstract] OR nefazodone[Title/Abstract] OR trazodone[Title/Abstract] OR vilazodone[Title/Abstract] OR bupropion[Title/Abstract] OR reboxetine[Title/Abstract] OR agomelatine[Title/Abstract] OR noradrenergic and specific serotonergic antidepressant*[Title/Abstract] OR NaSSA[Title/Abstract] OR NaSSAs[Title/Abstract] OR mirtazapine[Title/Abstract] OR TCA[Title/Abstract] OR TCAs[Title/Abstract] OR tricyclic[Title/Abstract] OR amersergide[Title/Abstract] OR amineptine[Title/Abstract] OR amitriptyline[Title/Abstract] OR amoxapine[Title/Abstract] OR butriptyline[Title/Abstract] OR clomipramine[Title/Abstract] OR clorimipramine[Title/Abstract] OR demexiptiline[Title/Abstract] OR desipramine[Title/Abstract] OR dothiepin[Title/Abstract] OR doxepin[Title/Abstract] OR imipramine[Title/Abstract] OR lofepramine[Title/Abstract] OR melitracen[Title/Abstract] OR metapramine[Title/Abstract] OR nortriptyline[Title/Abstract] OR noxiptiline[Title/Abstract] OR opipramol[Title/Abstract] OR protriptyline[Title/Abstract] OR quinupramine[Title/Abstract] OR tianeptine[Title/Abstract] OR trimipramine[Title/Abstract]))AND "mixed"[Title/Abstract] AND ("depressive disorder"[MeSH Terms] OR "depressive disorders"[Title/Abstract] OR "disorder depressive"[Title/Abstract] OR "disorders depressive"[Title/Abstract] OR "neurosis depressive"[Title/Abstract] OR "depressive neuroses"[Title/Abstract] OR "depressive neurosis"[Title/Abstract] OR "neuroses depressive"[Title/Abstract] OR "depression endogenous"[Title/Abstract] OR "depressions endogenous"[Title/Abstract] OR "endogenous depression"[Title/Abstract] OR "endogenous depressions"[Title/Abstract] OR "depressive syndrome"[Title/Abstract] OR "depressive syndromes"[Title/Abstract] OR "syndrome depressive"[Title/Abstract] OR "syndromes depressive"[Title/Abstract] OR "depression neurotic"[Title/Abstract] OR (("depressed"[All Fields] OR "Depression"[MeSH Terms] OR "Depression"[All Fields] OR "Depressions"[All Fields] OR "depression s"[All Fields] OR "depressive disorder"[MeSH Terms] OR ("Depressive"[All Fields] AND "Disorder"[All Fields]) OR "depressive disorder"[All Fields] OR "depressivity"[All Fields] OR "Depressive"[All Fields] OR "depressively"[All Fields] OR "depressiveness"[All Fields] OR "depressives"[All Fields]) AND "Neurotic"[Title/Abstract]) OR "neurotic depression"[Title/Abstract] OR "neurotic depressions"[Title/Abstract] OR "Melancholia"[Title/Abstract] OR "Melancholias"[Title/Abstract] OR "unipolar depression"[Title/Abstract] OR "depression unipolar"[Title/Abstract] OR "depressions unipolar"[Title/Abstract] OR "unipolar depressions"[Title/Abstract] OR "depressive episode"[Title/Abstract] OR "depressive episodes"[Title/Abstract])

**Detailed evaluation criteria of the risk of bias assessment**

Cochrane Collaboration’s tool was used for DB-RCTs (Higgins et al., 2011), the Newcastle-Ottawa Quality Assessment Scale (NOS) was used to evaluate the methodological quality of observational studies (with control groups) and post hoc analyses (Wells et al., 2013), an adapted version of the Quality Assessment Tool for Before-After (Pre-Post) Studies with No Control Group from the National Institutes of Health (NIH) was used for observational studies with no control group (*Study Quality Assessment Tools | NHLBI, NIH, (n.d.). Https://Www.Nhlbi.Nih.Gov/Health-Topics/Study-Quality-Assessment-Tools (Accessed August 4, 2020).*, n.d.), JBI was used for case series studies (Munn et al., 2019), and the Risk of Bias In Non-randomized Studies of Interventions (ROBINS) was used for open-label studies (Sterne et al., 2016).

A. For DB-RCTs, we used Cochrane Collaboration’s tool:

| Selection bias | | Performance bias | Detection bias | Attrition bias | Reporting bias | Other bias |
| --- | --- | --- | --- | --- | --- | --- |
| Random sequence generation | Allocation concealment | Blinding of participants and personnel | Blinding of outcome assessment | Incomplete outcome data | Selective reporting | Anything else, ideally prespecified |

1. For observational studies (with control group) and post-hoc analysis, we used the Newcastle-Ottawa Quality Assessment Scale (NOS):

| Selection | | | | Comparability | Outcome | | | Total  scores |
| --- | --- | --- | --- | --- | --- | --- | --- | --- |
| Representativeness  of the exposed  cohort | Selection of the  non exposed  cohort | Ascertainm  ent of  exposure | Demonstration that  outcome of interest  was not present at  start of study | Comparability of  cohorts on the basis  of the design or  analysis | Assessment of  outcome | Was follow-up long  enough for outcomes  to occur | Adequacy of  follow up of  cohorts | Full score  is 9 points |
| 1 | 1 | 1 | 1 | 2 | 1 | 1 | 1 | 9 |

0-3 points: low quality; 4-6 points: moderate quality; 7-9 points: high quality

C. For the observational studies with no control group, we used an adapted version of the Quality Assessment Tool for Before-After (Pre-Post) Studies with No Control Group from the National Institutes of Health (NIH):

| Criteria | Yes/No/Other(CD, NR, NA)* |
| --- | --- |
| 1. Was the study question or objective clearly stated? |  |
| 2. Were eligibility/selection criteria for the study population prespecified and clearly described? |  |
| 3. Were the participants in the study representative of those who would be eligible for the test/service/intervention in the general or clinical population of interest? |  |
| 4. Were all eligible participants that met the prespecified entry criteria enrolled? |  |
| 5. Was the sample size sufficiently large to provide confidence in the findings? |  |
| 6. Was the test/service/intervention clearly described and delivered consistently across the study population? |  |
| 7. Were the outcome measures prespecified, clearly defined, valid, reliable, and assessed consistently across all study participants? |  |
| 8. Were the people assessing the outcomes blinded to the participants' exposures/interventions? |  |
| 9. Was the loss to follow-up after baseline 20% or less? Were those lost to follow-up accounted for in the analysis? |  |
| 10. Did the statistical methods examine changes in outcome measures from before to after the intervention? Were statistical tests done that provided p values for the pre-to-post changes? |  |
| 11. Were outcome measures of interest taken multiple times before the intervention and multiple times after the intervention (i.e., did they use an interrupted time-series design)? |  |
| 12. If the intervention was conducted at a group level (e.g., a whole hospital, a community, etc.) did the statistical analysis take into account the use of individual-level data to determine effects at the group level? |  |

*CD, cannot determine; NA, not applicable; NR, not reported

Calculate the number of “ Yes” as the total score and evaluate the quality based on total score: a score of 0-4 represents a poor grade, 5-8 represents a fair grade, and 9-12 represents a good grade.

D. For case series, we used JBI critical appraisal tool:

| Question | Yes | No | Unclear | Not applicable |
| --- | --- | --- | --- | --- |
| 1. Were there clear criteria for inclusion in the case series? |  |  |  |  |
| 2. Was the condition measured in a standard, reliable way for all participants included in the case series? |  |  |  |  |
| 3. Were valid methods used for identification of the condition for all participants included in the case series? |  |  |  |  |
| 4. Did the case series have consecutive inclusion of participants? |  |  |  |  |
| 5. Did the case series have complete inclusion of participants? |  |  |  |  |
| 6. Was there clear reporting of the demographics of the participants in the study? |  |  |  |  |
| 7. Was there clear reporting of clinical information of the participants? |  |  |  |  |
| 8. Were the outcomes or follow-up results of cases clearly reported? |  |  |  |  |
| 9. Was there clear reporting of the presenting sites’/clinics’ demographic information? |  |  |  |  |
| 10. Was statistical analysis appropriate? |  |  |  |  |

Overall appraisal: Include □ Exclude □ Seek further info □

Comments (Including reason for exclusion)

____________________________________________________________________________________________________________________

E. For open-label study, we uesd Risk Of Bias In Non-randomized Studies of Interventions (ROBINS):

| Domain | Explanation |
| --- | --- |
| Domain1: Bias due to confounding | Baseline confounding occurs when one or more prognostic variables (factors that predict the outcome of interest) also predicts the intervention received at baseline ROBINS-I can also address time-varying confounding, which occurs when individuals switch between the interventions being compared and when post-baseline prognostic factors affect the intervention received after baseline. |
| Domain2: Bias in selection of participants into the study | When exclusion of some eligible participants, or the initial follow-up time of some participants, or some outcome events is related to both intervention and outcome, there will be an association between interventions and outcome even if the effects of the interventions are identical This form of selection bias is distinct from confounding—A specific example is bias due to the inclusion of prevalent users, rather than new users, of an intervention. |
| Domain3: Bias in classification of interventions | Bias introduced by either differential or non-differential misclassification of intervention status Non-differential misclassification is unrelated to the outcome and will usually bias the estimated effect of intervention towards the null Differential misclassification occurs when misclassification of intervention status is related to the outcome or the risk of the outcome, and is likely to lead to bias. |
| Domain4: Bias due to deviations from intended interventions | Bias that arises when there are systematic differences between experimental intervention and comparator groups in the care provided, which represent a deviation from the intended intervention(s) Assessment of bias in this domain will depend on the type of effect of interest (either the effect of assignment to intervention or the effect of starting and adhering to intervention). |
| Domain5: Bias due to missing data | Bias that arises when later follow-up is missing for individuals initially included and followed (such as differential loss to follow-up that is affected by prognostic factors); bias due to exclusion of individuals with missing information about intervention status or other variables such as confounders |
| Domain6: Bias in measurement of outcomes | Bias introduced by either differential or non-differential errors in measurement of outcome data. Such bias can arise when outcome assessors are aware of intervention status, if different methods are used to assess outcomes in different intervention groups, or if measurement errors are related to intervention status or effects. |

(Continued.)

| Domain | Explanation |
| --- | --- |
| Domain7:Bias in selection of the reported result | Selective reporting of results in a way that depends on the findings and prevents the estimate from being included in a meta-analysis (or other synthesis) |

F. Interpretation of domain-level and overall risk of bias judgements in ROBINS-I:

| Judgement | Within each domain | Across domains | Criterion |
| --- | --- | --- | --- |
| Low risk of bias | The study is comparable to a well performed randomised trial with regard to this domain | The study is comparable to a well performed randomised trial | The study is judged to be at low risk of bias for all domains |
| Moderate risk of bias | The study is sound for a non-randomised study with regard to this domain but cannot be considered comparable to a well performed randomised trial | The study provides sound evidence for a nonrandomised study but cannot be considered comparable to a well performed randomised trial | The study is judged to be at low or moderate risk of bias for all domains |
| Serious risk of bias | The study has some important problems in this domain | The study has some important problems | The study is judged to be at serious risk of bias in at least one domain, but not at critical risk of bias in any domain |
| Critical risk of bias | The study is too problematic in this domain to provide any useful evidence on the effects of intervention | The study is too problematic to provide any useful evidence and should not be included in any synthesis | The study is judged to be at critical risk of bias in at least one domain |
| No information | No information on which to base a judgement about risk of bias for this domain | No information on which to base a judgement about risk of bias | There is no clear indication that the study is at serious or critical risk of bias and there is a lack of information in one or more key domains of bias (a judgement is required for this) |

**Reference**

Higgins, J. P. T., Altman, D. G., Gotzsche, P. C., Juni, P., Moher, D., Oxman, A. D., … Cochrane Statistical Methods Group. (2011). The Cochrane Collaboration’s tool for assessing risk of bias in randomised trials. *BMJ*, *343*(oct18 2), d5928–d5928. https://doi.org/10.1136/bmj.d5928

Munn, Z., Barker, T. H., Moola, S., Tufanaru, C., Stern, C., McArthur, A., … Aromataris, E. (2019). Methodological quality of case series studies: An introduction to the JBI critical appraisal tool. *JBI Database of Systematic Reviews and Implementation Reports*, *Publish Ahead of Print*. https://doi.org/10.11124/JBISRIR-D-19-00099

Sterne, J. A., Hernán, M. A., Reeves, B. C., Savović, J., Berkman, N. D., Viswanathan, M., … Higgins, J. P. (2016). ROBINS-I: A tool for assessing risk of bias in non-randomised studies of interventions. *BMJ*, i4919. https://doi.org/10.1136/bmj.i4919

*Study Quality Assessment Tools | NHLBI, NIH, (n.d.). Https://www.nhlbi.nih.gov/health-topics/study-quality-assessment-tools (accessed August 4, 2020).* (n.d.).

Wells, Shea, O’Connell, Peterson, Welch, Losos, & Tugwell. (2013). *Wells, G. A., Shea, B., O’Connell, D., Peterson, J. B., Welch, V., Losos, M., & Tugwell, P. (2013). The Newcastle-Ottawas Scale (NOS) for assessging the quality of non-randomized studies in meta-analyses. Retrieved from http://www.ohri. Ca/programs/clinical_epidemiology/oxford.asp.*

**Quality assessment**

Among the 24 included studies, four were DB-RCTs, one was an open-label study, 11 were post hoc analyses, seven were retrospective studies (two with a control group and five without a control group), and one was a case series study.

Due to the low risk in all items of the three DB-RCTs, the overall risk of bias was rated as low. The other RCT did not report the use of the allocation concealment method, and it had a small sample size (N = 56) and a high loss to follow-up rate (58.9%), thus, we considered that the overall risk of bias of this RCT was relatively high. The risk of bias in the open-label study was serious. For the observational studies with control groups and post hoc analyses, seven studies were rated as high quality while six were rated as moderate quality. Four observational studies without a control group were rated as moderate quality and one was rated as poor quality. The case series study was finally included in the present study after careful evaluation.

**Supplementary Table 1** Characteristics of included studies.

| Study | Age (yrs) | Female (%) | Treatment (dose) | Definition | Study design | Sample size | Duration | Outcome measures | Results |
| --- | --- | --- | --- | --- | --- | --- | --- | --- | --- |
| **Major depressive disorder - Antipsychotics** | | | | | | | | | |
| Suppes et al. (2016)  Post hoc studies  *Clayton et al. (2018)*  *Pikalov et al. (2017)*  *Goldberg et al. (2020)*  *Goldberg et al. (2017)* | LUR:  43.6±12.1  PBO:  46.4±12.0 | LUR:67%  PBO:72% | LUR  (20-60 mg/d) | MDE (DSM-IV) with 2-3 manic symptoms | PBO-controlled  DB-RCT | n=109 LUR  n=100 PBO | 6 weeks | MADRS score reduction  YMRS score reduction  CGI score reduction  SDS score reduction  MADRS response  (≥ 50% reduction)  MADRS remission  (total score ≤ 12) | LUR > PBO  LUR > PBO  LUR > PBO  LUR > PBO  LUR > PBO  LUR > PBO |
| Patkar et al. (2012)  Post hoc study  *Pae et al. (2012)* | ZIP:  39.1±11.9  PBO:  38.7±12.7 | ZIP: 52.9%  PBO: 52.6% | ZIP  (80-160 mg/d) | MDE of BD II or MDD (DSM-IV) with 2-3  manic symptoms | PBO-controlled  DB-RCT | n=35 ZIP  (15 with MDD)  n=38 PBO  (14 with MDD) | 6 weeks | MADRS score reduction  YMRS score reduction  CGI-BP score reduction  Response  Remission | ZIP > PBO  ZIP = PBO  ZIP = PBO  ZIP > PBO  ZIP > PBO |

BD, II bipolar disorder type II; CGI, Clinical Global Impressions Scale; CGI-BP, Clinical Global Impressions Scale for Bipolar Disorder; DB-RCT, double-blind, randomized placebo-controlled trial; DSM, diagnostic and statistical manual of mental disorders; LUR, lurasidone; MADRS, Montgomery-Asberg Depression Rating scale; MDD, major depressive disorder; MDE, major depressive episodes; PBO, placebo; SDS, Sheehan Disability Scale; YMRS, Young Mania Rating Scale; ZIP, ziprasidone.

**Supplementary Table 1** (Continued).

| Study | Age (yrs) | Female (%) | Treatment (dose) | Definition | Study design | Sample size | Duration | Outcome measures | Results |
| --- | --- | --- | --- | --- | --- | --- | --- | --- | --- |
| Durgam et al. (2025) | LUM:  44.0±15.0  PBO:  45.0±14.8 | LUM: 59.8%  PBO: 59.1% | LUM  (42mg/d) | MDE with at least 3  manic/hypomanic symptoms (DSM-5) | PBO-controlled  DB-RCT | n=92 LUM  n=93 PBO | 6 weeks | MADRS score reduction  YMRS score reduction  CGI-S score reduction  MADRS response  MADRS remission | LUM > PBO  LUM > PBO  LUM > PBO  LUM > PBO  LUM > PBO |
| Han et al. (2019) | 28 | 34.2% | ARI  (4.0±0.8 mg/d) | MDE with at least 3  manic/hypomanic symptoms (DSM-5) | Retrospective study  (with no control group) | n=38 ARI | 8 weeks | MADRS score reduction  CGI-S score reduction  YMRS score reduction  SDS score reduction | -7. 1, p <0.0001  -0.8, p <0.0001  -4.9, p <0.0001  -4. 1, p <0.0001 |
| **Major depressive disorder - Antidepressants** | | | | | | | | | |
| Serro et al. (2019) | 54.74 | 75.0% | TRZ  (50-300mg/) | MDE with at least 3  manic/hypomanic symptoms (DSM-5) | Retrospective study  (with no control group) | n=32 TRZ | 5±2 days | MRS score reduction  CGI score reduction  MADRS score reduction | -7.1 p <0.001;  -1.19, p <0.001;  -10.55, p <0.001. |
| Carmellini et al. (2025) | 50.05±15.84 | 59.8% | IV TRZ  (Mean dose = 92.13mg/d) | MDE with at least 3  manic/hypomanic symptoms (DSM-5) | Retrospective study  (with no control group) | n=97 IV TRZ | 8.51 days | MADRS response  MADRS-item 3  score reduction  MADRS-item 4  score reduction | 51.54%  -3.19, p <0.001;  -3.12, p <0.001; |

ARI, aripiprazole; CGI-S, Clinical Global Impression-Severity; IV TRZ, intravenous trazodone; LUM, lumateperone; MRS, Mania Rating Scale; TRZ, trazodone.

**Supplementary Table 1** (Continued).

| Study | Age (yrs) | Female (%) | Treatment (dose) | Definition | Study design | Sample size | Duration | Outcome measures | Results |
| --- | --- | --- | --- | --- | --- | --- | --- | --- | --- |
| **Major depressive disorder - Mood stabilizer** | | | | | | | | | |
| Liu (2014) | 51.7±13.4 | 68.2% | VAL  (day 1: 100-250 mg/d;  endpoint: 100- 1250  mg/d) | MDD with mixed depressive  features (e.g., irritability,  hostility, talkativeness, and distractibility) | Retrospective, case-  series observational  study | n=22 VAL | 3-60 months | Treatment responses  (markedly improved,  Moderately improved,  minimal/no change, worsened) | Markedly improved: n=9;  moderately  improved: n=9;  minimal/no change: n=4. |
| **Bipolar disorder - Antipsychotics** | | | | | | | | | |
| Benazzi et al. (2009) | OLZ:  41.0±12.4  PBO:  40.3±12.0 | OLZ=61.3%,  PBO=62.1% | OLZ  (OLZ: 5-20 mg/d) | BD I, in a current  MDE (DSM-IV) with ≥ 2 manic/hypomanic symptoms | Post hoc  analysis of  PBO-controlled  DB-RCT | n=173 OLZ  n=166 PBO | 8 weeks | Response rate  Switch rate (YMRS ≥ 15) | OLZ > PBO  OLZ = PBO |
| Patkar et al. (2012)  Post hoc study  *Pae et al.* (2012) | ZIP:  39.1±11.9  PBO:  38.7±12.7 | ZIP: 52.9%  PBO: 52.6% | ZIP  (80-160 mg/d) | MDE of BD II or MDD (DSM-IV) with 2-3  manic symptoms | PBO-controlled  DB-RCT | n=35 ZIP  (15 with MDD)  n=38 PBO  (14 with MDD) | 6 weeks | MADRS score reduction  YMRS score reduction  CGI-BP score reduction  Response  Remission | ZIP > PBO  ZIP = PBO  ZIP = PBO  ZIP > PBO  ZIP > PBO |

BD I, bipolar disorder type I; OLZ, olanzapine; OFC, the olanzapine/fluoxetine combination; VAL, valproate.

**Supplementary Table 1** (Continued).

| Study | Age (yrs) | Female (%) | Treatment (dose) | Definition | Study design | Sample size | Duration | Outcome measures | Results |
| --- | --- | --- | --- | --- | --- | --- | --- | --- | --- |
| Durgam et al. (2025) | LUM:  42.0±14.3  PBO:  41.0±12.9 | LUM: 64.0%  PBO: 64.0% | LUM  (42mg/d) | Bipolar depression with at least 3  manic/hypomanic symptoms (DSM-5) | PBO-controlled  DB-RCT | n=100 LUM  n=100 PBO | 6 weeks | MADRS score reduction  YMRS score reduction  CGI-S score reduction  MADRS response  MADRS remission | LUM > PBO  LUM > PBO  LUM > PBO  LUM = PBO  LUM > PBO |
| Mclntyre et al. (2020) | PBO:  43.3±11.6  1.5 mg/d, CAR:  41.4±12. 1  3 mg/d, CAR:  42.4±11.4 | PBO: 61. 1%,  1.5 mg/d;  CAR: 62.9%,  3 mg/d;  CAR: 60. 1% | CAR  (1.5 or 3.0 mg/d) | BD I, with a current MDE (DSM-IV or DSM-5) with YMRS ≥ 4 | Post hoc  analysis of  PBO-controlled  DB-RCT | n=275  1.5mg/d CAR  n=271  3.0mg/d CAR  n=262 PBO | 6 weeks | MADRS score reduction  YMRS score reduction  MADRS response  MADRS remission | CAR > PBO for both doses  CAR =PBO for both doses  CAR > PBO for both doses  CAR > PBO for both doses |
| Mclntyre et al. (2023) | LUM:  46.4±13.3  PBO:  45.4±12.5 | LUM: 60.3%  PBO: 67.5% | LUM  (42 mg/d) | BD I or BD II, with a current MDE (DSM-5) with YMRS score ≥ 4 and ≤12 | Post hoc  analysis of  PBO-controlled  DB-RCT | n=73 LUM  n=83 PBO | 6 weeks | MADRS score reduction  YMRS score reduction  CGI-BP-S mania  subscore reduction | LUM > PBO  LUM = PBO  LUM = PBO |

CAR, cariprazine; CGI-BP-S, Clinical Global Impression Scale-Bipolar Version-Severity.

**Supplementary Table 1** (Continued).

| Study | Age (yrs) | Female (%) | Treatment (dose) | Definition | Study design | Sample size | Duration | Outcome measures | Results |
| --- | --- | --- | --- | --- | --- | --- | --- | --- | --- |
| Mclntyre et al. (2015) | LUR:  42.2±11.8  PBO:  40.7±11.5 | LUR: 63.2%;  PBO: 55.6% | LUR  (20-60 mg/d  or 80-120 mg/d) | BD I, with a current MDE (DSM-IV or DSM-5) with YMRS score ≥ 4 | Post hoc  analysis of  PBO-controlled  DB-RCT | n=182 LUR  n=90 PBO | 6 weeks | MADRS score reduction  YMRS score reduction  MADRS response  MADRS remission | LUR > PBO  LUR = PBO  LUR > PBO  LUR > PBO |
| Singh et al. (2020) | LUR:  13.6±2.2  PBO:  14.1±2.2 | LUR: 44.3%  PBO: 39.3% | LUR  (20-80 mg/d) | BD I, with a current MDE (DSM-5), 10- 17 years of age, with  YMRS score ≥ 5 | Post hoc  analysis of  PBO-controlled  DB-RCT | n=97 LUR  n=89 PBO | 6 weeks | CDRS-R score reduction  Treatment-emergent  hypomania or mania | LUR > PBO  LUR = PBO |
| Tohen et al. (2014) | 39.1±12.6 | 63.2% | OLZ  (Study 1: 5-20 mg/d  Study 2: 5-20 mg/d) | BD I, with MADRS score ≥ 20  (Study 1)/HAM-D score ≥ 18  (Study 2), with ≥ 3 concurrent  manic symptoms | Post hoc  analysis of  two PBO-controlled  DB-RCTs | n=204 OLZ  n=166 PBO | 6 weeks | MADRS score reduction  YMRS score reduction  MADRS response  MADRS remission | OLZ > PBO  OLZ > PBO  OLZ = PBO  OLZ > PBO |
| **Bipolar disorder - Antidepressants** | | | | | | | | | |
| Benazzi et al. (2009) | OLZ:  41.0±12.4  OFC:  36.4±12.6 | OLZ=61.3%,  OFC=73.0% | OLZ(5-20 mg/d)  OFC  (OFC: 6/25,6/50, or 12/50 mg/d) | BD I, in a current  MDE (DSM-IV) with ≥ 2 manic/hypomanic symptoms | Post hoc  analysis of  PBO-controlled  DB-RCT | n=173 OLZ  n=37 OFC | 8 weeks | Response rate  Switch rate (YMRS ≥ 15) | OLZ = PBO  OLZ = PBO |

CDRS-R, the Children’s Depression Rating Scale-Revised;

**Supplementary Table 1** (Continued).

| Study | Age (yrs) | Female (%) | Treatment (dose) | Definition | Study design | Sample size | Duration | Outcome measures | Results |
| --- | --- | --- | --- | --- | --- | --- | --- | --- | --- |
| Goldberg et al. (2007) | with AD:  40.3±11.2  without AD:  38.3±11.1 | / | AD | BD I, with a current MDE (DSM-IV) with ≥ 2 manic symptoms | Naturalistic,  observational  study | n=145 with AD  n=190 without AD | 3 months | Time to recovery (CMF ≤ 2 affective symptoms for ≥ 8 weeks) or state of recovering (CMF ≤ 2 affective symptoms for ≥ 4 weeks) | with AD = without AD |
| **Bipolar disorder - Mood stabilizers** | | | | | | | | | |
| Amodeo et al. (2017) | 47.78 | 58.0% | ISV  (day 1: 592 mg/d; following  days: 800 mg/d) | BD, in a current MDE with at least 3  manic/hypomanic symptoms (DSM-5) | Retrospective  study (with no  control group) | n=50 ISV | 3.72 days | Change in CGI-BP  score | The mean CGI-BP score decreased , with a reduction of 45.95% in the score of manic symptoms |
| Buoli et al. (2021) | IV-VAL:  44.22±14.56  IV-DEL:  50.41±14.54 | IV-VAL:  78.4%  IV-DEL:  75.0% | IV-VAL (100-400 mg/d)  IV-DEL  (0.5-2.0 mg/d) | BD, in a current MDE with at least 3  manic/hypomanic symptoms (DSM-5) | Open-label study | n=51 IV-VAL n=32 IV-DEL | 5 days | MADRS score reduction  YMRS score reduction  Response rate | IV-VAL >IV-DEL  IV-VAL > IV-DEL  IV-VAL > IV-DEL |
| Federico et al. (2019) | 21-84 | 59.1% | Oral and intravenous VAL | BD, in a current MDE with at least 3  manic/hypomanic symptoms (DSM-5) | Retrospective  study | n=21 oral VAL  n=45 intravenous  VAL | 3 days  (T1-T3) | MADRS score reduction  YMRS score reduction | -8.0 vs. -7.0  -5.0 vs. -6.0 |

AD, adjunctive antidepressants; CMF, Clinical Monitoring Form; ISV, intravenous sodium valproate; IV-DEL, intravenous delorazepam; IV-VAL, intravenous valproic acid.

**Supplementary Table 1** (Continued).

| Study | Age (yrs) | Female (%) | Treatment (dose) | Definition | Study design | Sample size | Duration | Outcome measures | Results |
| --- | --- | --- | --- | --- | --- | --- | --- | --- | --- |
| Santucci et al. (2019) | 16-85 | 60.5% | ISV  (400- 1200mg/d) | BD I and BD II, in a manic or depressive episode with mixed features (DSM-5) | Retrospective observational study (with no control group) | n=119 ISV | 3.8 days  (T0:baseline;  T1: switch to oral therapy) | CGI-S score reduction  MADRS score reduction  YMRS score reduction | T0=6.2; T1=3.6  T0=34.4; T1=21.6  T0=23; T1=16.8 |
| Wang et al. (2023) | QTP:  22. 14±7.44  QTP+V:  20. 18±4.42  QTP+L:  22.40±8.30 | QTP:  48.85%  QTP+V:  72.7%  QTP+L:  60.0% | QTP:  300-600 mg/day  QTP+V:  Valproate:  500-1000mg/day  QTP+L:  Lithium: 600-900 mg/day | BD I or II, in a MDE (DSM-5) with 2-3 manic symptoms | Randomized controlled pilot study | n= 35 QTP  n=11 QTP+V  n=10 QTP+L | Phase 1:  All received QTP for 2 weeks;  Phase 2  (randomization):  6 weeks | MADRS score reduction  YMRS score reduction  CUDOS-M score reduction | QTP+L:  -7.18, p=0.025  QTP+V:  -8.6, p=0.027  QTP+L:  -4.82, p=0.047  QTP+V:  -0.23, p=0.936  QTP+L:  -5.41, p=0.071  QTP+V:  -1.84, p=0.605 |

CUDOS-M, Clinically Useful Depression Outcome Scale supplemented with questions for the DSM-5 mixed features specifier; QTP, quetiapine; QTP+L, quetiapine plus lithium; QTP+V, quetiapine plus valproate.

**Included studies**

Amodeo, G., Olivola, M., & Fagiolini, A. (2017). Intravenous sodium valproate for the treatment of mixed symptoms in patients with bipolar disorder: A pilot study. *European Neuropsychopharmacology*, *27*, S857–S858. https://doi.org/10.1016/S0924-977X(17)31540-7

Benazzi, F., Berk, M., Frye, M. A., Wang, W., Barraco, A., & Tohen, M. (2009). Olanzapine/Fluoxetine Combination for the Treatment of Mixed Depression in Bipolar I Disorder: A Post Hoc Analysis. *The Journal of Clinical Psychiatry*, *70*(10), 1424–1431. https://doi.org/10.4088/JCP.08m04772gre

Buoli, M., Ceresa, A., Barkin, J. L., Weston, C., Mucci, F., Pozzoli, S., & Esposito, C. M. (2021). Intravenous Augmentative Valproate Versus Delorazepam in Bipolar Patients With a Major Depressive Mixed Episode and Partial/Nonresponse to Oral Treatment: A Pilot Study. *Journal of Clinical Psychopharmacology*, *41*(1), 79–81. https://doi.org/10.1097/JCP.0000000000001314

Carmellini, P., Cuomo, A., Pierini, C., Pardossi, S., Pinzi, M., Mariantoni, E., & Fagiolini, A. (2025). Intravenous trazodone for the treatment of psychomotor agitation and associated symptoms in major depressive disorder patients experiencing a depressive episode with mixed features. *International Clinical Psychopharmacology*. https://doi.org/10.1097/YIC.0000000000000580

Clayton, A. H., Tsai, J., Mao, Y., Pikalov, A., & Loebel, A. (2018). Effect of Lurasidone on Sexual Function in Major Depressive Disorder Patients With Subthreshold Hypomanic Symptoms (Mixed Features): Results From a Placebo-Controlled Trial. *The Journal of Clinical Psychiatry*, *79*(5). https://doi.org/10.4088/JCP.18m12132

Durgam, S., Kozauer, S. G., Earley, W. R., Chen, C., Huo, J., Lakkis, H., … McIntyre, R. S. (2025). Lumateperone for the Treatment of Major Depressive Disorder With Mixed Features or Bipolar Depression With Mixed Features: A Randomized Placebo-Controlled Trial. *Journal of Clinical Psychopharmacology*, *45*(2), 67–75. https://doi.org/10.1097/JCP.0000000000001964

Federico, E., Baldini, I., Salvaro, F., Desantis, S., Del Matto, L., Olivola, M., … Fagiolini, A. (2019). P.056 Treatment of mixed states: A comparison between oral and intravenous valproate. *European Neuropsychopharmacology*, *29*, S58. https://doi.org/10.1016/j.euroneuro.2019.09.118

Goldberg, J. F., Ng-Mak, D., Siu, C., Chuang, C.-C., Rajagopalan, K., & Loebel, A. (2017). Remission and recovery associated with lurasidone in the treatment of major depressive disorder with subthreshold hypomanic symptoms (mixed features): Post-hoc analysis of a randomized, placebo-controlled study with longer-term extension. *CNS Spectrums*, *22*(2), 220–227. https://doi.org/10.1017/S1092852917000025

Goldberg, J. F., Perlis, R. H., Ghaemi, S. N., Calabrese, J. R., Bowden, C. L., Wisniewski, S., … Thase, M. E. (2007). Adjunctive Antidepressant Use and Symptomatic Recovery Among Bipolar Depressed Patients With Concomitant Manic Symptoms: Findings From the STEP-BD. *American Journal of Psychiatry*, *164*(9), 1348–1355. https://doi.org/10.1176/appi.ajp.2007.05122032

Goldberg, J. F., Siu, C., Mao, Y., Tsai, J., Pikalov, A., Calabrese, J. R., & Loebel, A. (2020). Major depressive disorder with mixed features and treatment response to lurasidone: A symptom network model. *Journal of Affective Disorders*, *277*, 1045–1054. https://doi.org/10.1016/j.jad.2020.08.048

Han, C., Wang, S.-M., Bahk, W.-M., Lee, S.-J., Patkar, A. A., Masand, P. S., & Pae, C.-U. (2019). The Potential Utility of Aripiprazole Augmentation for Major Depressive Disorder with Mixed Features Specifier: A Retrospective Study. *Clinical Psychopharmacology and Neuroscience*, *17*(4), 495–502. https://doi.org/10.9758/cpn.2019.17.4.495

Liu, C.-C. (2014). Adjuvant valproate therapy for patients with suspected mixed-depressive features. *Therapeutic Advances in Psychopharmacology*, *4*(4), 143–148. https://doi.org/10.1177/2045125314532868

McIntyre, R. S., Cucchiaro, J., Pikalov, A., Kroger, H., & Loebel, A. (2015). Lurasidone in the Treatment of Bipolar Depression With Mixed (Subsyndromal Hypomanic) Features: Post Hoc Analysis of a Randomized Placebo-Controlled Trial. *The Journal of Clinical Psychiatry*, *76*(04), 398–405. https://doi.org/10.4088/JCP.14m09410

McIntyre, R. S., Durgam, S., Huo, J., Kozauer, S. G., & Stahl, S. M. (2023). The Efficacy of Lumateperone in Patients With Bipolar Depression With Mixed Features. *The Journal of Clinical Psychiatry*, *84*(3). https://doi.org/10.4088/JCP.22m14739

McIntyre, R. S., Suppes, T., Earley, W., Patel, M., & Stahl, S. M. (2020). Cariprazine efficacy in bipolar I depression with and without concurrent manic symptoms: Post hoc analysis of 3 randomized, placebo-controlled studies. *CNS Spectrums*, *25*(4), 502–510. https://doi.org/10.1017/S1092852919001287

Pae, C.-U., Patkar, A., Gilmer, W., Holtzman, N., Thommi, S., & Ghaemi, S. (2012). Predictors of Response to Ziprasidone: Results from a 6-Week Randomized Double-Blind, Placebo-Controlled Trial for Acute Depressive Mixed State. *Pharmacopsychiatry*, *45*(04), 152–155. https://doi.org/10.1055/s-0031-1297984

Patkar, A., Gilmer, W., Pae, C., Vöhringer, P. A., Ziffra, M., Pirok, E., … Ghaemi, S. N. (2012). A 6 week randomized double-blind placebo-controlled trial of ziprasidone for the acute depressive mixed state. *PloS One*, *7*(4), e34757.

Pikalov, A., Goldberg, J., Siu, C., Mao, Y., Tsai, J., & Loebel, A. (2017). Lurasidone for the treatment of major depressive disorder with mixed features: Do manic symptoms moderate treatment response? *European Neuropsychopharmacology*, *27*, S831–S832. https://doi.org/10.1016/S0924-977X(17)31499-2

Santucci, A., Amodeo, G., Fagiolini, A., & Goracci, A. (2019). Treatment of mixed states using intravenous sodium valproate: Efficacy and tolerability. *European Neuropsychopharmacology*, *29*, S56–S57. https://doi.org/10.1016/j.euroneuro.2018.11.1032

Serro, V. L., Crapanzano, C., Goracci, A., Bolognesi, S., Cicco, G. D., Cini, E., … Fagiolini, A. (2019). P.290 Early efficacy of trazodone for the treatment of depressive episodes with mixed features in patients with major depressive disorder. *European Neuropsychopharmacology*, *29*, S212–S213. https://doi.org/10.1016/j.euroneuro.2019.09.319

Singh, M. K., Pikalov, A., Siu, C., Tocco, M., & Loebel, A. (2020). Lurasidone in Children and Adolescents with Bipolar Depression Presenting with Mixed (Subsyndromal Hypomanic) Features: *Post Hoc* Analysis of a Randomized Placebo-Controlled Trial. *Journal of Child and Adolescent Psychopharmacology*, *30*(10), 590–598. https://doi.org/10.1089/cap.2020.0018

Suppes, T., Silva, R., Cucchiaro, J., Mao, Y., Targum, S., Streicher, C., … Loebel, A. (2016). Lurasidone for the Treatment of Major Depressive Disorder With Mixed Features: A Randomized, Double-Blind, Placebo-Controlled Study. *American Journal of Psychiatry*, *173*(4), 400–407. https://doi.org/10.1176/appi.ajp.2015.15060770

Tohen, M., Kanba, S., McIntyre, R. S., Fujikoshi, S., & Katagiri, H. (2014). Efficacy of olanzapine monotherapy in the treatment of bipolar depression with mixed features. *Journal of Affective Disorders*, *164*, 57–62. https://doi.org/10.1016/j.jad.2014.04.003

Wang, Z., Zhang, D., Du, Y., Wang, Y., Huang, T., Ng, C. H., … Hu, S. (2023). Efficacy of Quetiapine Monotherapy and Combination Therapy for Patients with Bipolar Depression with Mixed Features: A Randomized Controlled Pilot Study. *Pharmaceuticals (Basel, Switzerland)*, *16*(2).

**Supplementary Table 2** The risk of bias of DB-RCTs was assessed by the Cochrane Collaboration’s tool.

| **Author**  **Year** | **Random sequence generation**  **(selection bias)** | **Allocation concealment**  **(selection bias)** | **Blinding of participants and personnel**  **(performance bias)** | **Blinding of outcome assessment**  **(detection bias)** | **Incomplete**  **Outcome data**  **(attrition bias)** | **Selective reporting**  **(reporting bias)** | **Other bias** | **Overall**  **Risk**  **Of**  **Bias** |
| --- | --- | --- | --- | --- | --- | --- | --- | --- |
| Suppes  2016 | Low | Low | Low | Low | Low | Low | Low | Low |
| Patkar  2012 | Low | Low | Low | Low | Low | Low | Low | Low |
| Wang  2023 | Low | Unclear | Low | Low | Low | Low | High | High |
| Durgam  2025 | Low | Low | Low | Low | Low | Low | Low | Low |

**Supplementary Table 3** The risk of bias of open-label study was assessed by the Risk Of Bias In Non-randomized Studies of Interventions (ROBINS).

| **Author**  **Year** | **Domain 1:**  **Confounding**  **factors** | **Domain 2:**  **Selection of**  **participants** | **Domain 3:**  **Intervention**  **classification** | **Domain 4:**  **Deviation from**  **intervention** | **Domain 5:**  **Missing data** | **Domain 6:**  **Measurement**  **of outcome** | **Domain 7:**  **Selection of**  **reported result** | **ROBINS-I**  **overall score** |
| --- | --- | --- | --- | --- | --- | --- | --- | --- |
| Buoli  2021 | Serious | Low | Moderate | Low | Moderate | Low | Moderate | Serious |

**Supplementary Table 4** The quality of observational studies (with control group) and post-hoc analysis was assessed by the Newcastle-Ottawa Quality Assessment Scale (NOS).

| **Author**  **Year** | **Selection** | | | | **Comparability** | **Outcome** | | | **Total scores** |
| --- | --- | --- | --- | --- | --- | --- | --- | --- | --- |
|  | **Representativeness of the exposed cohort** | **Selection of the non exposed cohort** | **Ascertainment of exposure** | **Demonstration that outcome of interest was not present at start of study** | **Comparability of cohorts on the basis of the design or analysis** | **Assessment of outcome** | **Was follow-up long enough for outcomes to occur** | **Adequacy of follow up of cohorts** |  |
| Clayton  2018 | ★ | ★ | ★ | ★ | ★☆ | ★ | ☆ | ☆ | 6 |
| Goldberg  2020 | ★ | ★ | ★ | ★ | ★☆ | ★ | ★ | ☆ | 7 |
| Goldberg  2017 | ★ | ★ | ★ | ★ | ★☆ | ★ | ★ | ☆ | 7 |
| Benazzi  2009 | ★ | ★ | ★ | ★ | ★☆ | ★ | ★ | ★ | 8 |
| Mclntyre  2020 | ☆ | ★ | ★ | ★ | ★☆ | ★ | ★ | ☆ | 6 |
| Mclntyre  2015 | ☆ | ★ | ★ | ★ | ★☆ | ★ | ★ | ★ | 7 |
| Singh  2020 | ☆ | ★ | ★ | ★ | ★★ | ★ | ★ | ★ | 8 |
| Tohen  2014 | ★ | ★ | ★ | ★ | ★☆ | ★ | ☆ | ☆ | 6 |
| Goldberg  2007 | ★ | ★ | ★ | ★ | ★★ | ★ | ★ | ☆ | 8 |
| Federico  2019 | ☆ | ★ | ★ | ★ | ☆☆ | ★ | ☆ | ☆ | 4 |
| Pikalov  2017 | ★ | ★ | ★ | ★ | ★☆ | ★ | ☆ | ☆ | 6 |

(Continued.)

| **Author**  **Year** | **Selection** | | | | **Comparability** | **Outcome** | | | **Total scores** |
| --- | --- | --- | --- | --- | --- | --- | --- | --- | --- |
|  | **Representativeness of the exposed cohort** | **Selection of the non exposed cohort** | **Ascertainment of exposure** | **Demonstration that outcome of interest was not present at start of study** | **Comparability of cohorts on the basis of the design or analysis** | **Assessment of outcome** | **Was follow-up long enough for outcomes to occur** | **Adequacy of follow up of cohorts** |  |
| Pae  2012 | ☆ | ★ | ★ | ★ | ☆☆ | ★ | ★ | ★ | 6 |
| Mclntyre  2023 | ☆ | ★ | ★ | ★ | ★★ | ★ | ★ | ★ | 8 |

0-3 points: low quality; 4-6 points: moderate quality; 7-9 points: high quality

**Supplementary Table 5** The quality of observational studies (with no control group) was assessed by the Quality Assessment Tool for Before-After (Pre-Post) Studies with No Control Group from the National Institutes of Health (NIH).

| **Author**  **Year** | **Q1** | **Q2** | **Q3** | **Q4** | **Q5** | **Q6** | **Q7** | **Q8** | **Q9** | **Q10** | **Q11** | **Q12** | **Total scores**  **(n=number of “Yes”)** | **Grading** |
| --- | --- | --- | --- | --- | --- | --- | --- | --- | --- | --- | --- | --- | --- | --- |
| Han  2019 | Yes | Yes | Yes | Yes | Yes | No | Yes | NR | NR | NR | NR | NA | 6 | Fair |
| Serro  2019 | Yes | Yes | Yes | NR | No | Yes | Yes | NR | NR | Yes | NR | NA | 6 | Fair |
| Santucci  2018 | Yes | Yes | Yes | NR | No | Yes | Yes | NR | Yes | No | NR | NA | 6 | Fair |
| Amodeo  2017 | Yes | No | Yes | No | No | Yes | No | NR | NR | NR | No | NA | 3 | Poor |
| Carmellini  2025 | Yes | Yes | No | CD | No | No | Yes | NR | NR | Yes | Yes | NA | 5 | Fair |

(Total scores 0-4=poor; 5-8=fair; 9-12=good)

(*CD:cannot determine; NA:not applicable; NR:not reported)

**Supplementary Table 6** The quality of case series was assessed by the JBI's critical appraisal tool.

| **Author**  **Year** | **Q1** | **Q2** | **Q3** | **Q4** | **Q5** | **Q6** | **Q7** | **Q8** | **Q9** | **Q10** | **Total Scores** |
| --- | --- | --- | --- | --- | --- | --- | --- | --- | --- | --- | --- |
| Liu2014 | Yes | Unclear | No | Yes | No | Yes | Yes | Yes | No | No | 5 |

Overall appraisal: Include


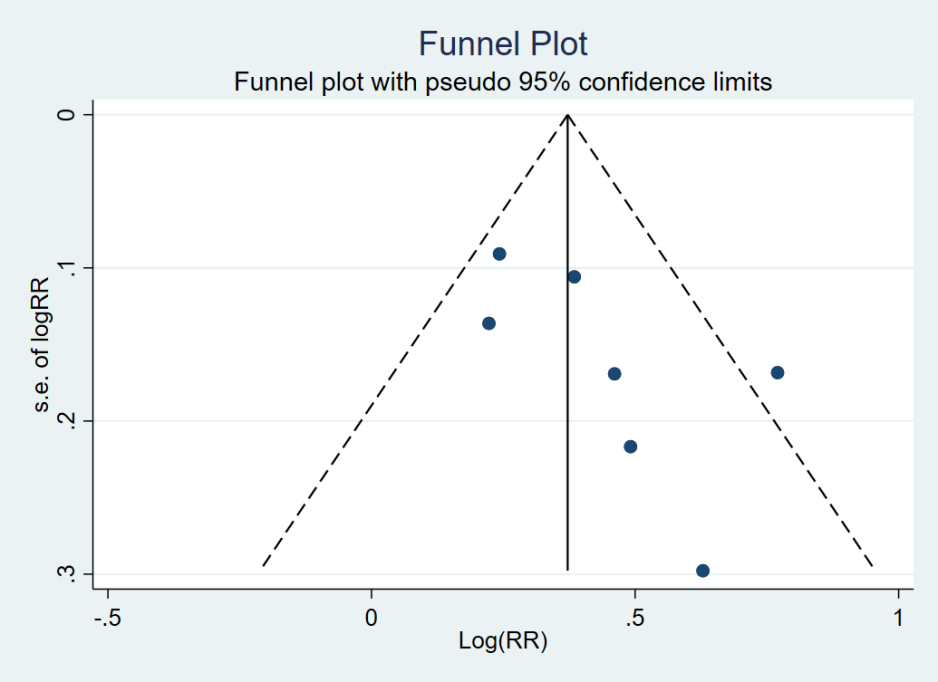


**Supplementary Figure 1** Funnel plot to detect the potential publication bias in the analysis of primary efficacy outcome on depressive symptoms (clinical response rate).


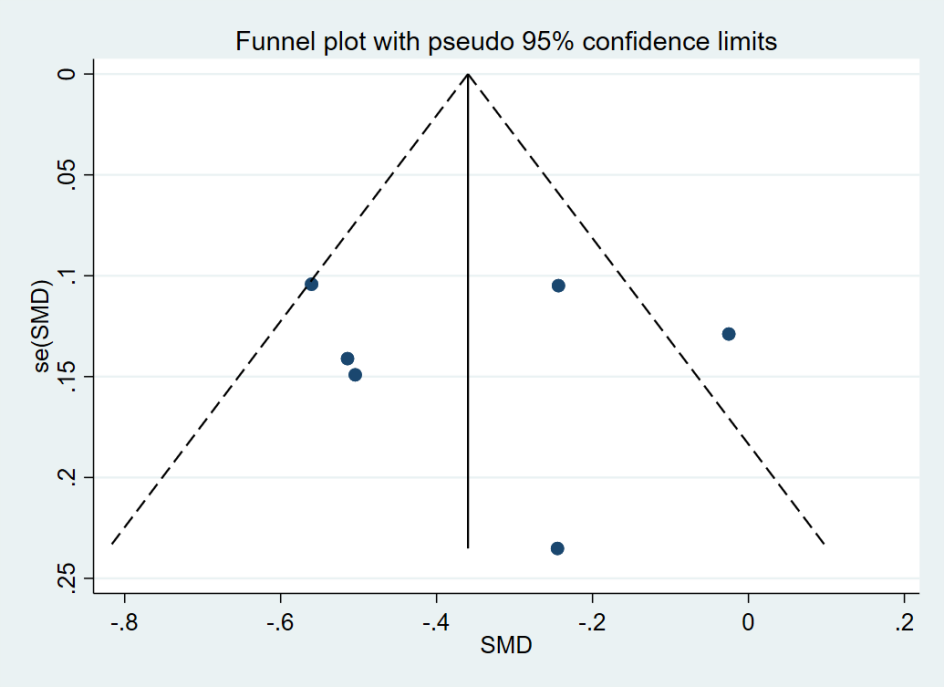


**Supplementary Figure 2** Funnel plot to detect the potential publication bias in the analysis of primary efficacy outcome on manic symptoms (changes in the YMRS score).


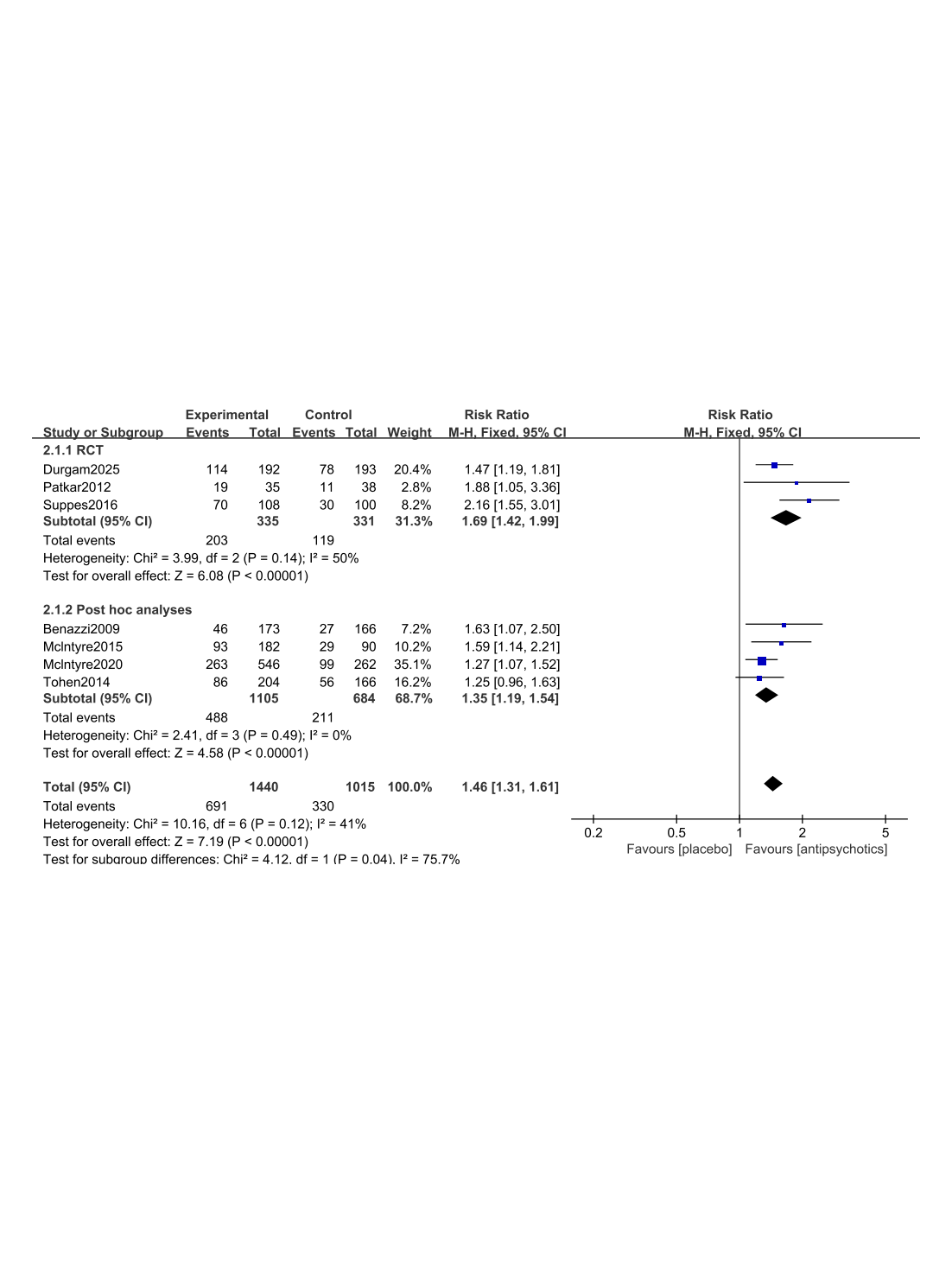


**Supplementary Figure 3** Forest plot for the clinical response rate of antipsychotics vs. placebo in subgroup analyses based on study design (randomized controlled trials vs. post-hoc analyses).


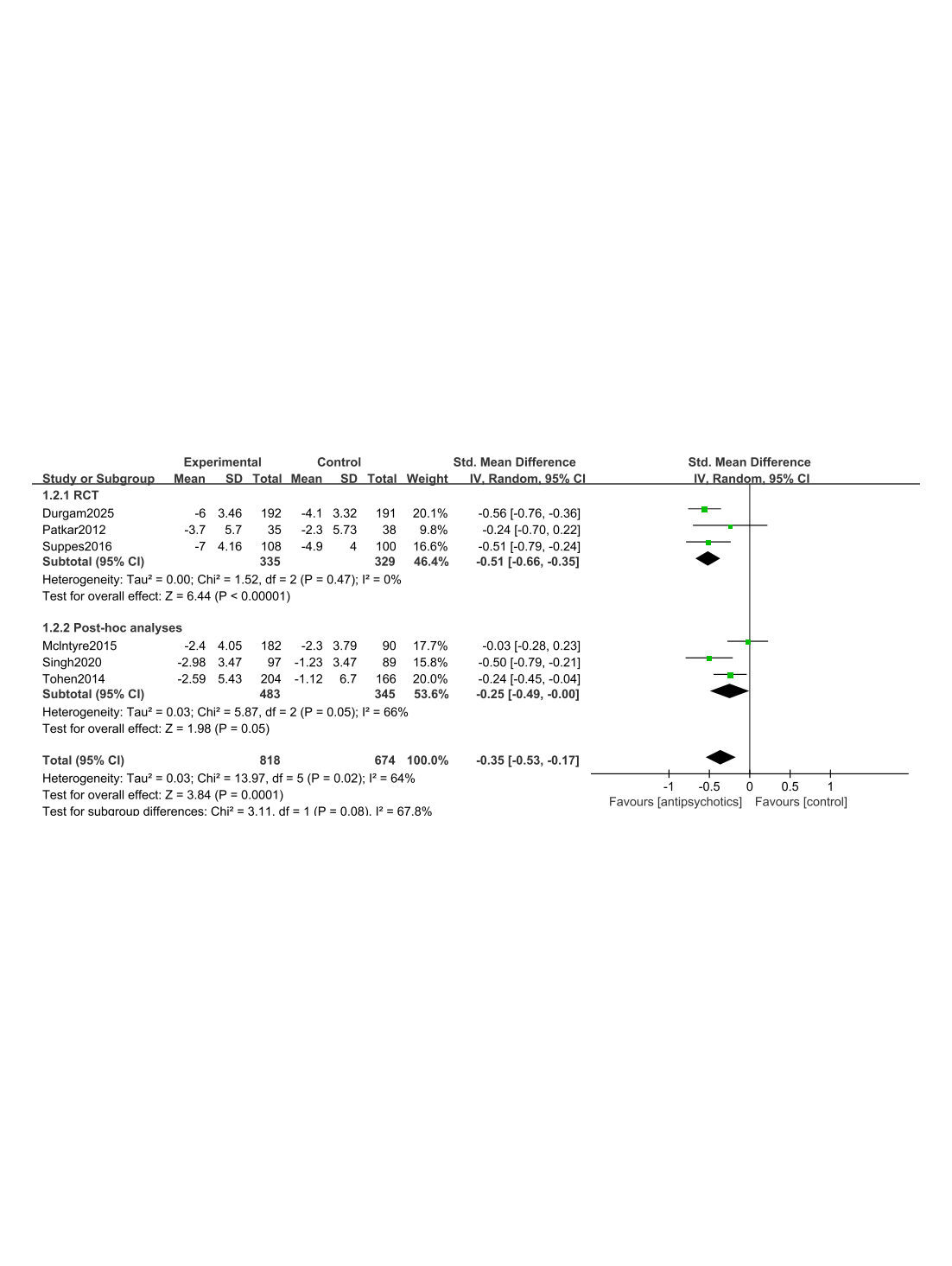


**Supplementary Figure 4** Forest plot for the changes in the YMRS score from baseline in patients with MDE-MFS treated with antipsychotics vs. placebo in subgroup analyses based on study design.


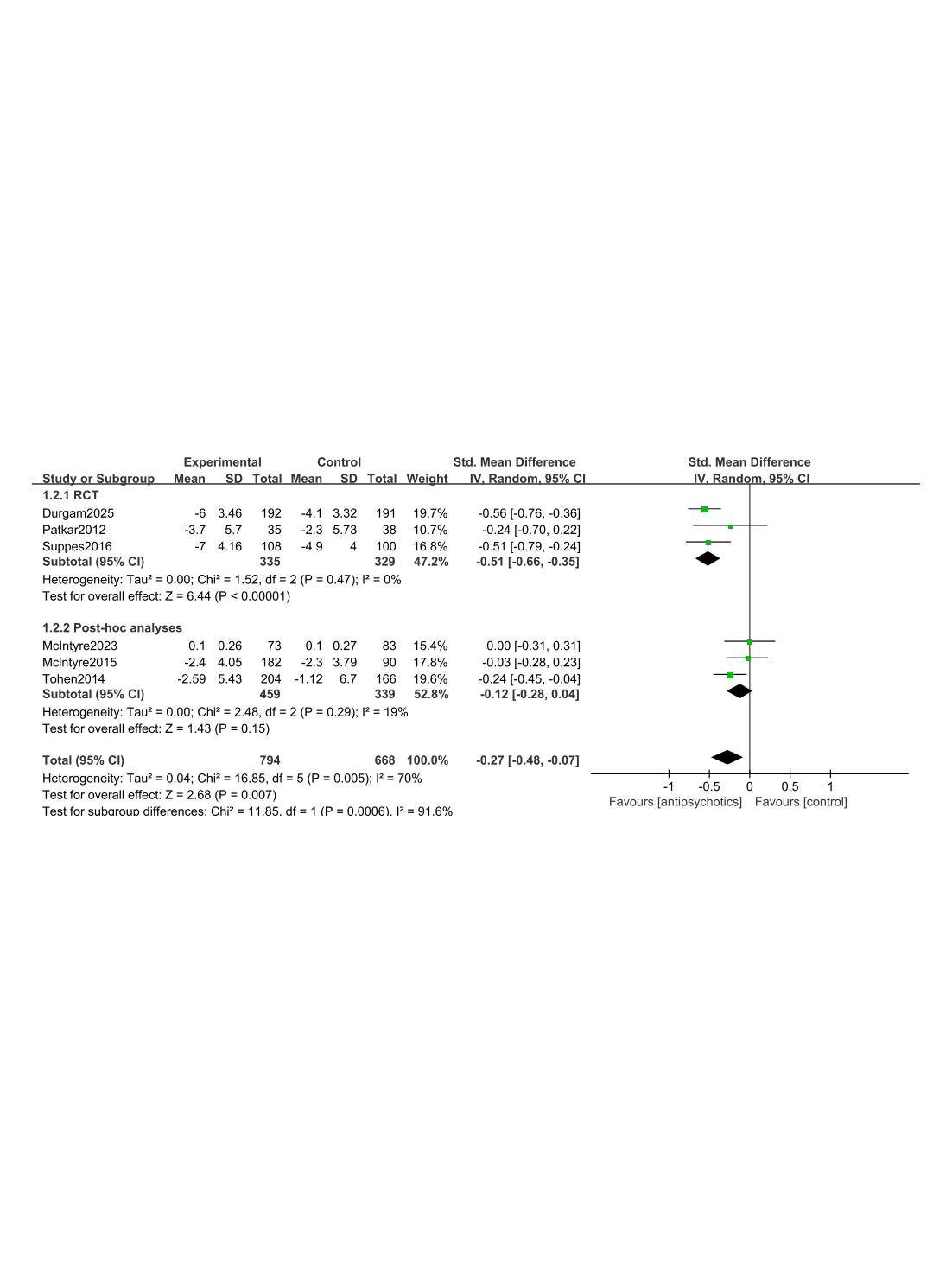


**Supplementary Figure 5** Forest plot for the changes in the YMRS score from baseline in patients with MDE-MFS treated with antipsychotics vs. placebo in subgroup analyses based on study design after excluding high heterogeneity study.


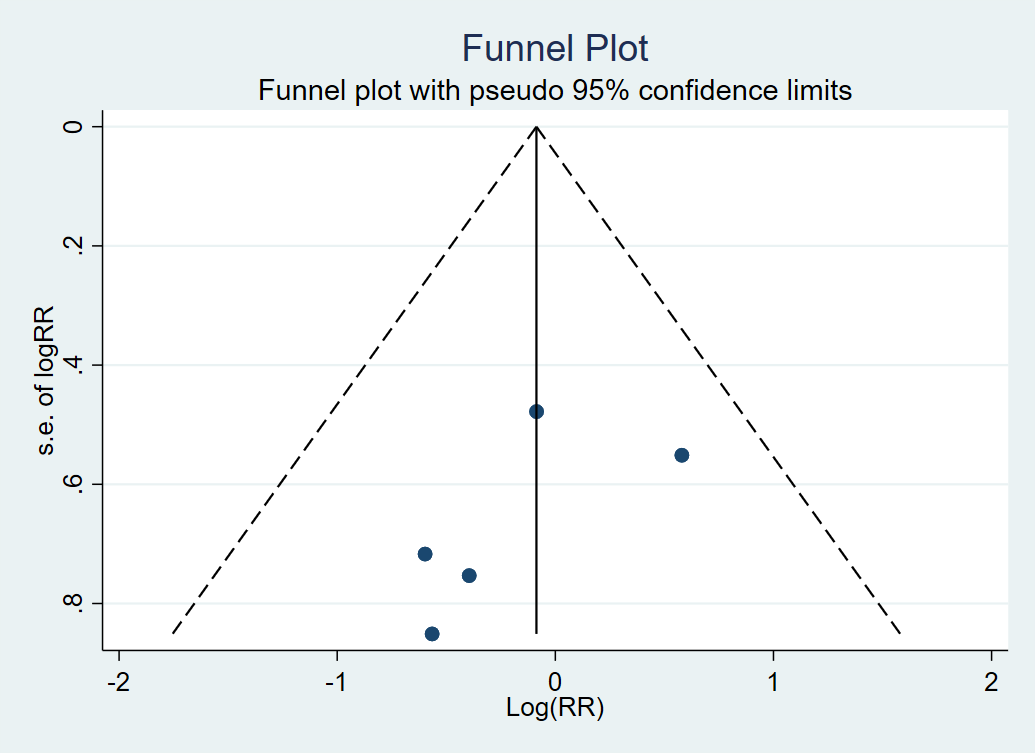


**Supplementary Figure 6** Funnel plot to detect the potential publication bias in the analysis of primary safety outcome.


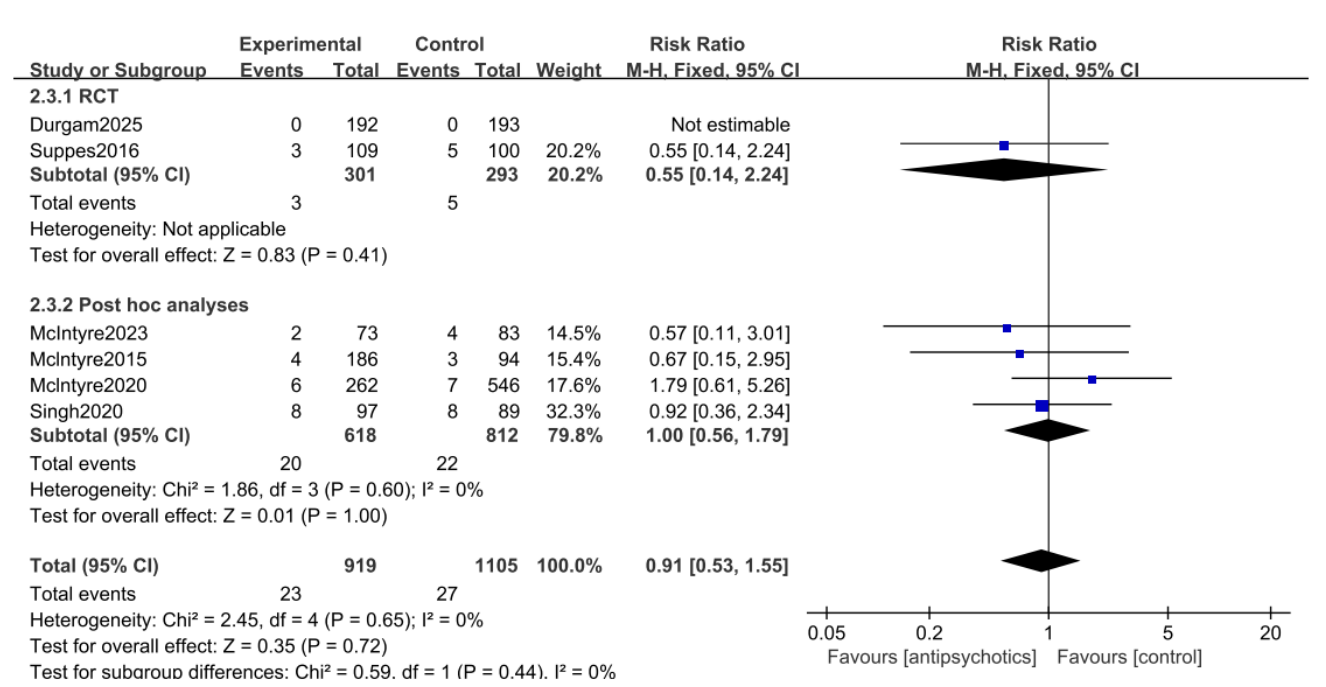


**Supplementary Figure 7** Forest plot for the rate of treatment-emergent hypomania/mania associated with antipsychotics vs. placebo in the treatment of MDE-MFS in subgroup analyses based on study design.


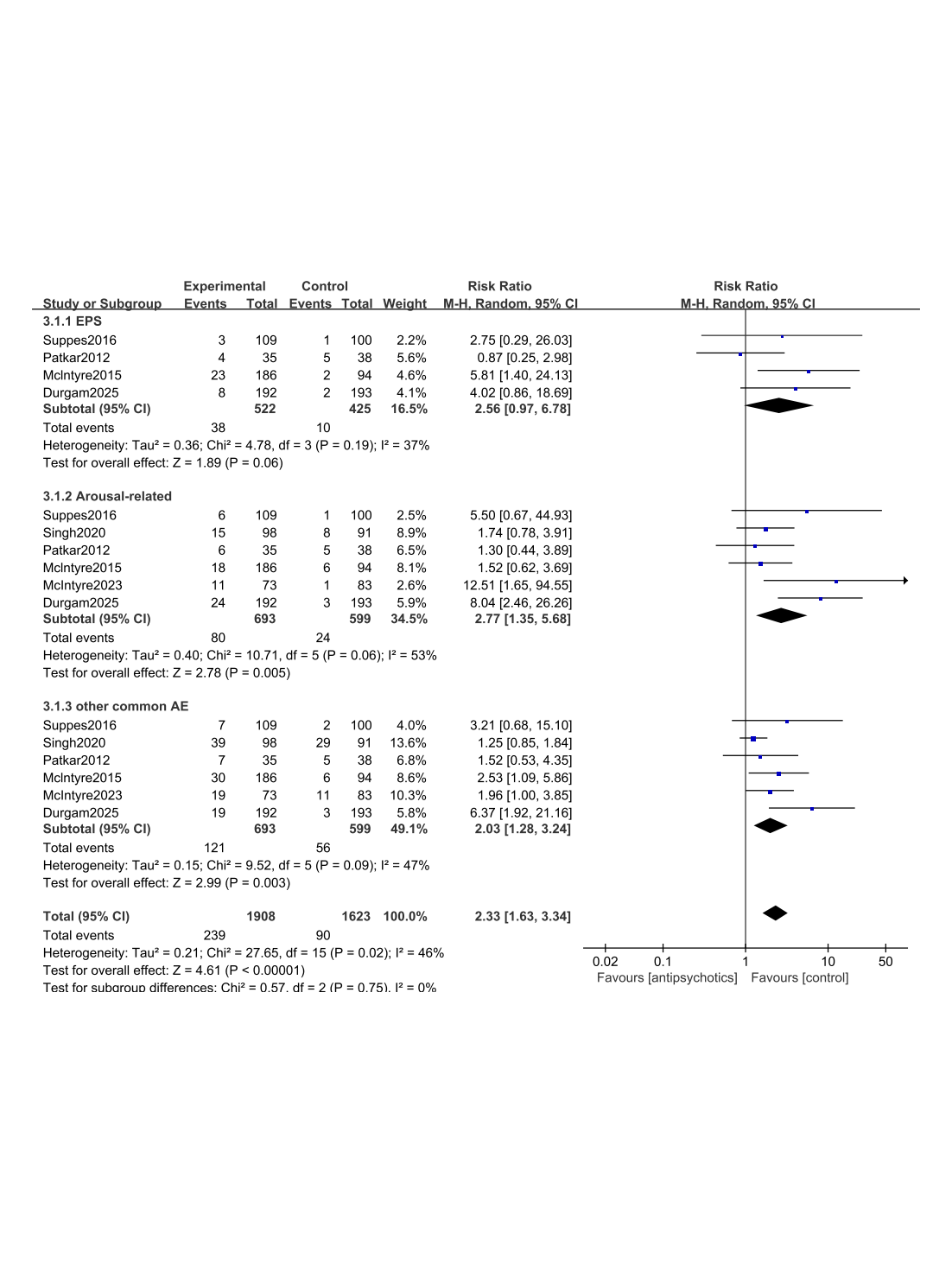


**Supplementary Figure 8** Forest plot for the risk of common adverse events between the antipsychotics and placebo in the treatment of MDE-MFS.


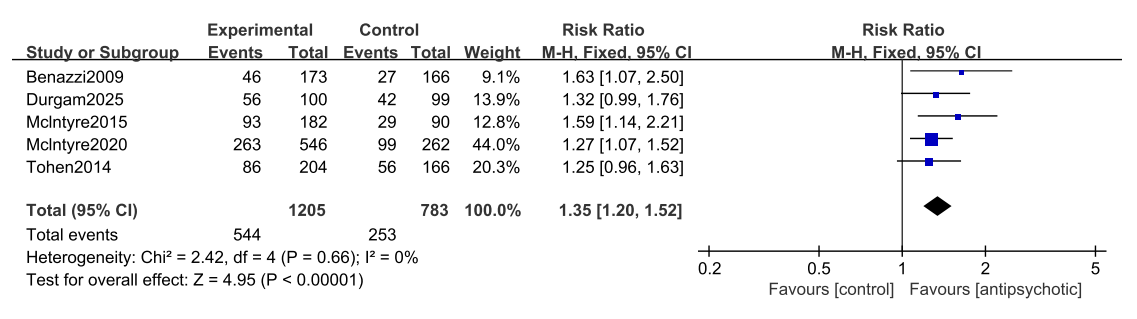


**Supplementary Figure 9** Forest plot for the clinical response rate of antipsychotics versus placebo in the treatment of MDE-MFS in bipolar disorder (BD).


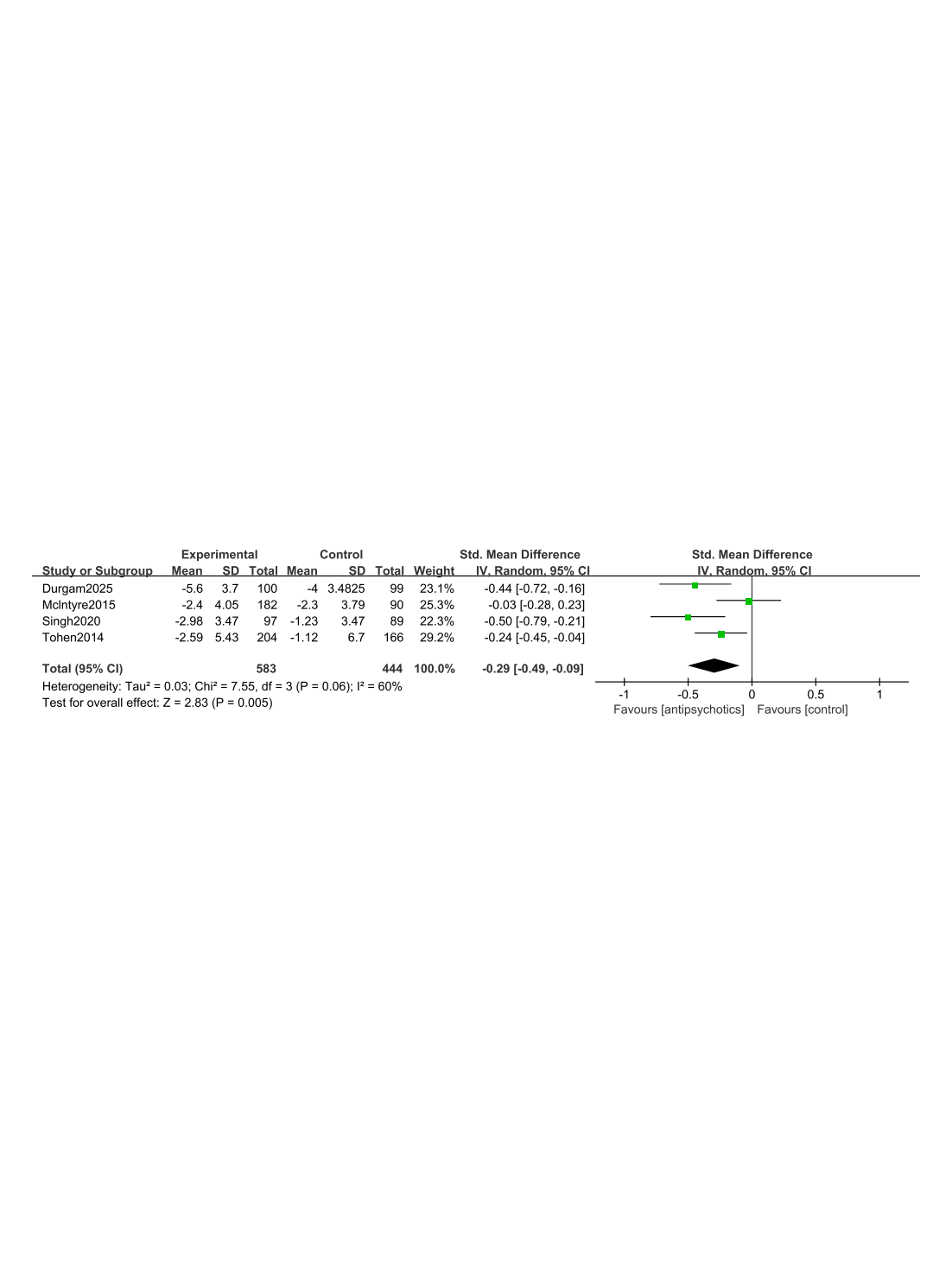


**Supplementary Figure 10** Forest plot for the changes in the YMRS score from baseline in BD patients with MDE-MFS treated with antipsychotics vs. placebo.


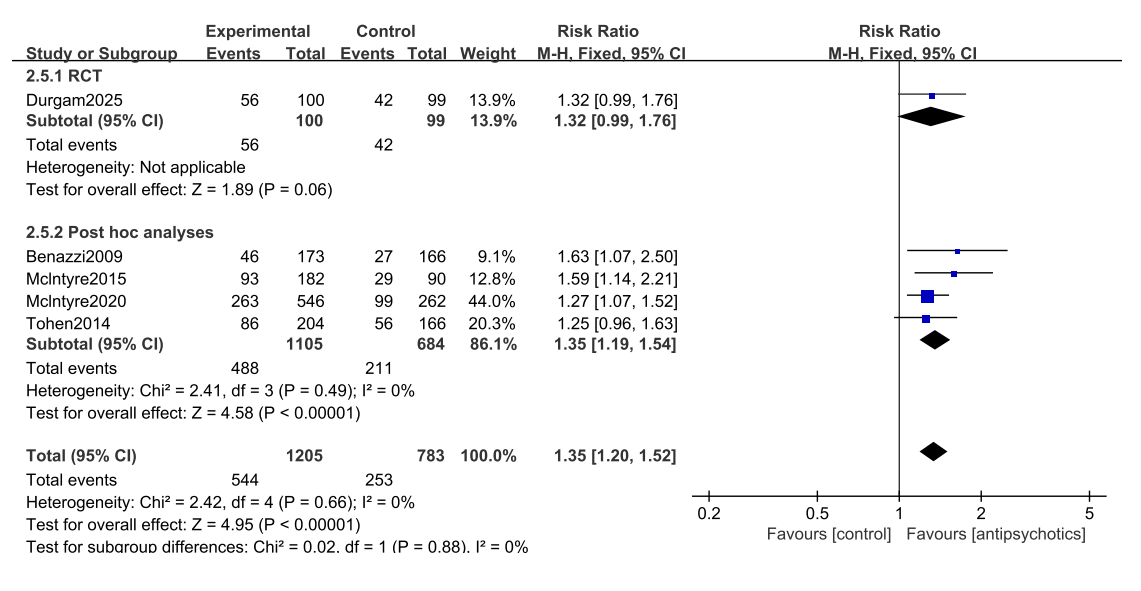


**Supplementary Figure 11** Forest plot for the clinical response rate of antipsychotics vs. placebo in BD patients with MDE-MFS in subgroup analyses based on study design.


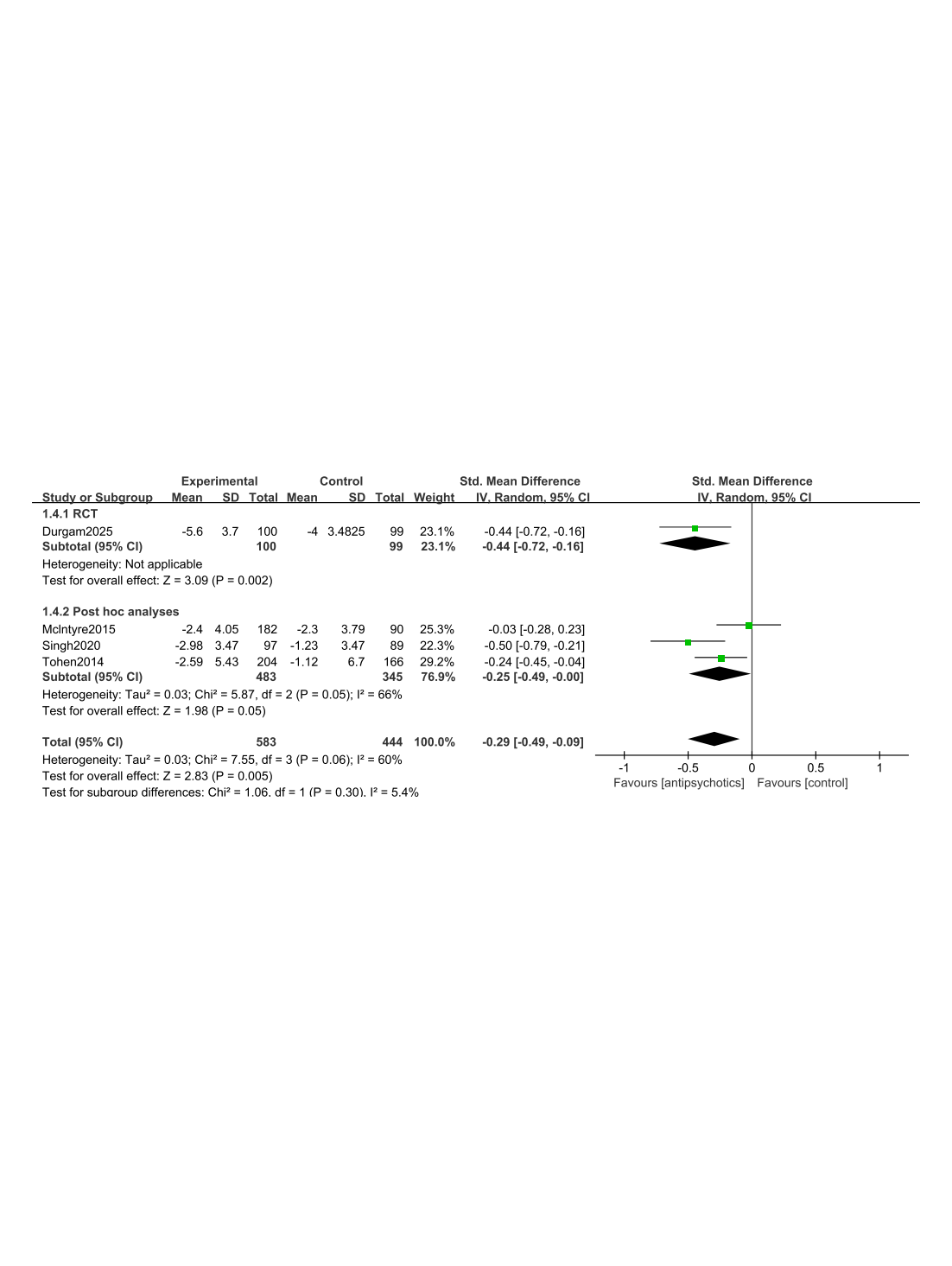


**Supplementary Figure 12** Forest plot for the changes in the YMRS score from baseline in BD patients with MDE-MFS treated with antipsychotics vs. placebo in subgroup analyses based on study design.


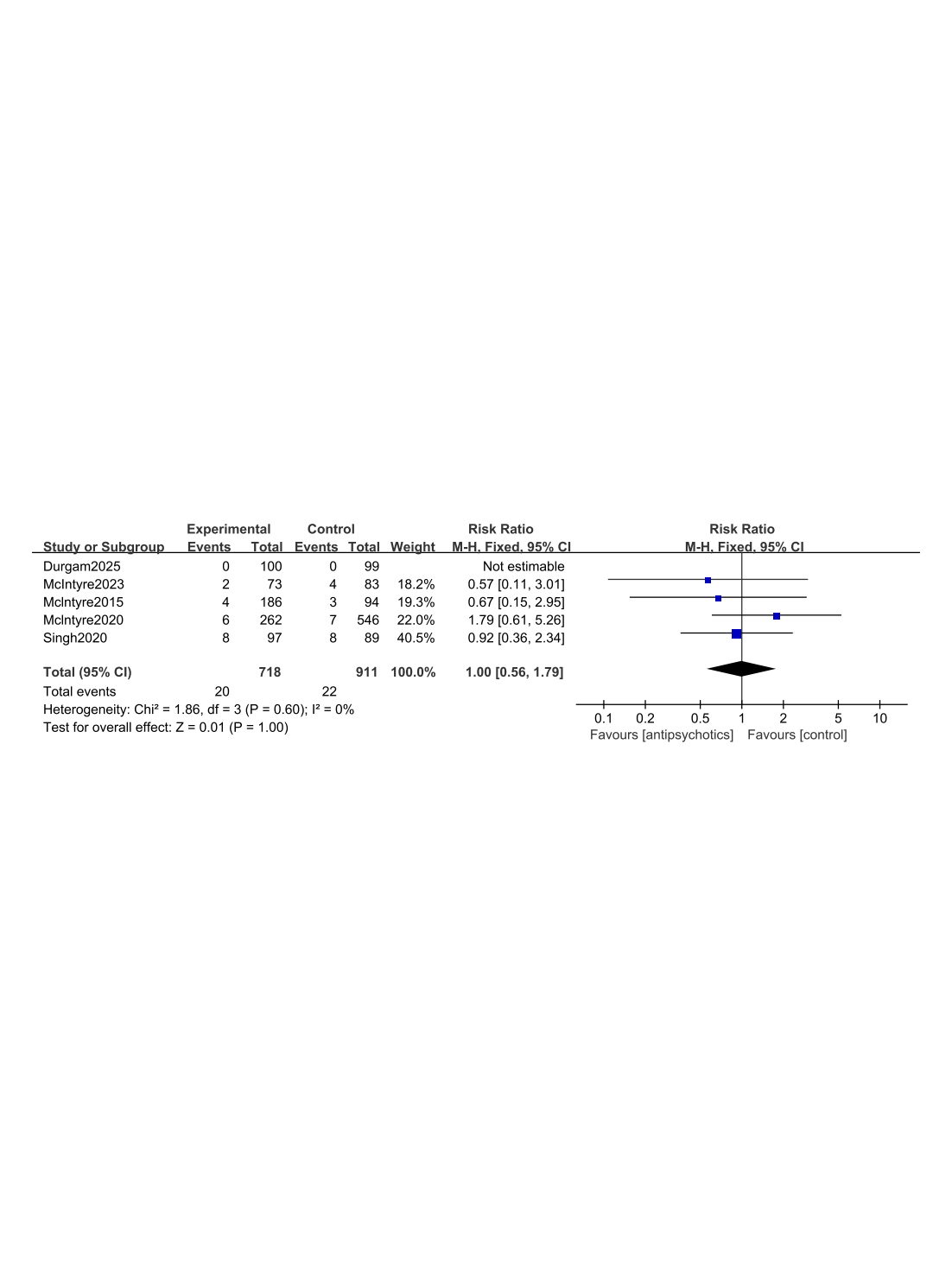


**Supplementary Figure 13** Forest plot for the rate of treatment-emergent hypomania/mania associated with antipsychotics vs. placebo in the treatment of BD patients with MDE-MFS.


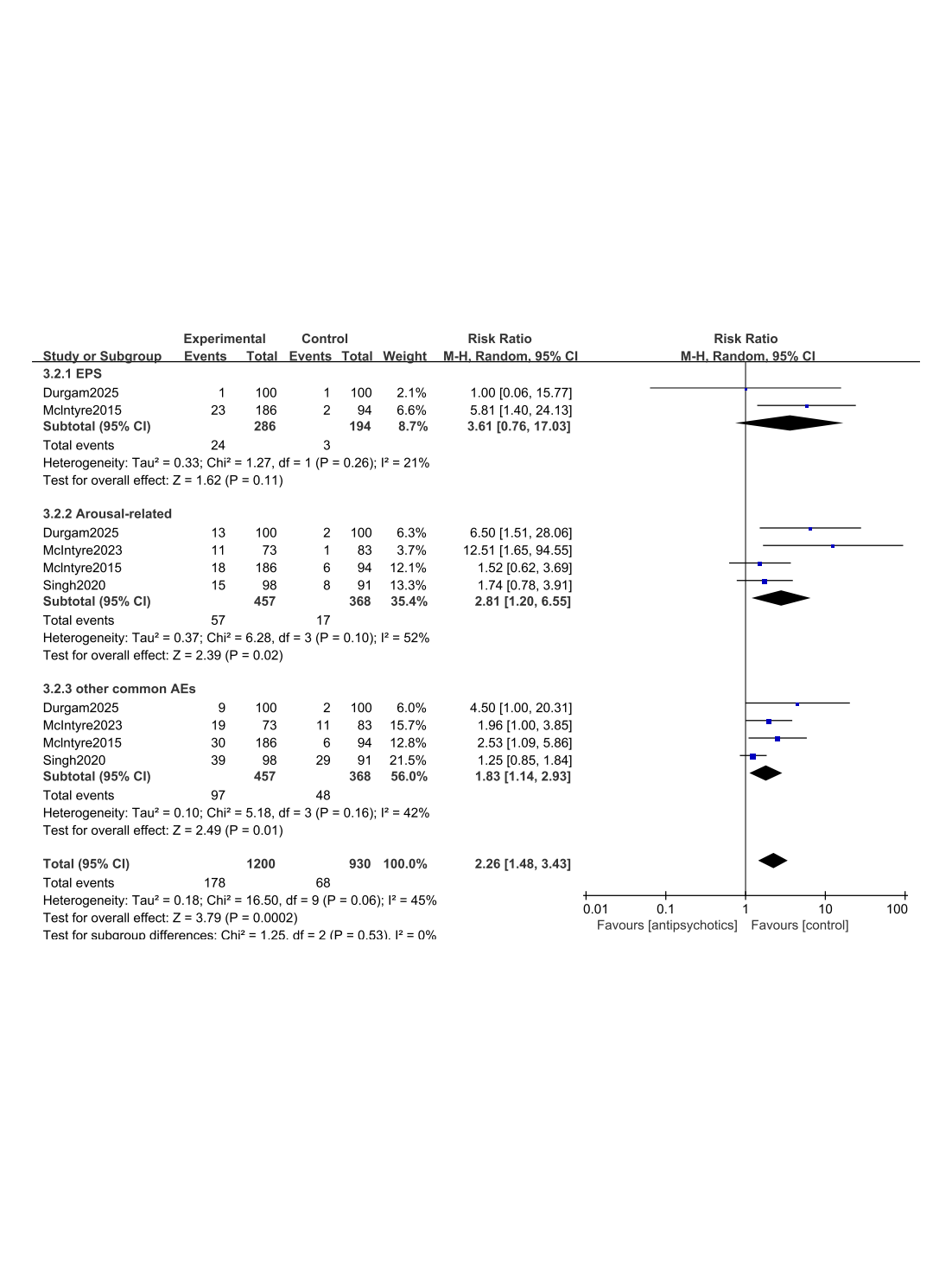


**Supplementary Figure 14** Forest plot for the risk of common adverse events between the antipsychotics and placebo in the treatment of BD patients with MDE-MFS.


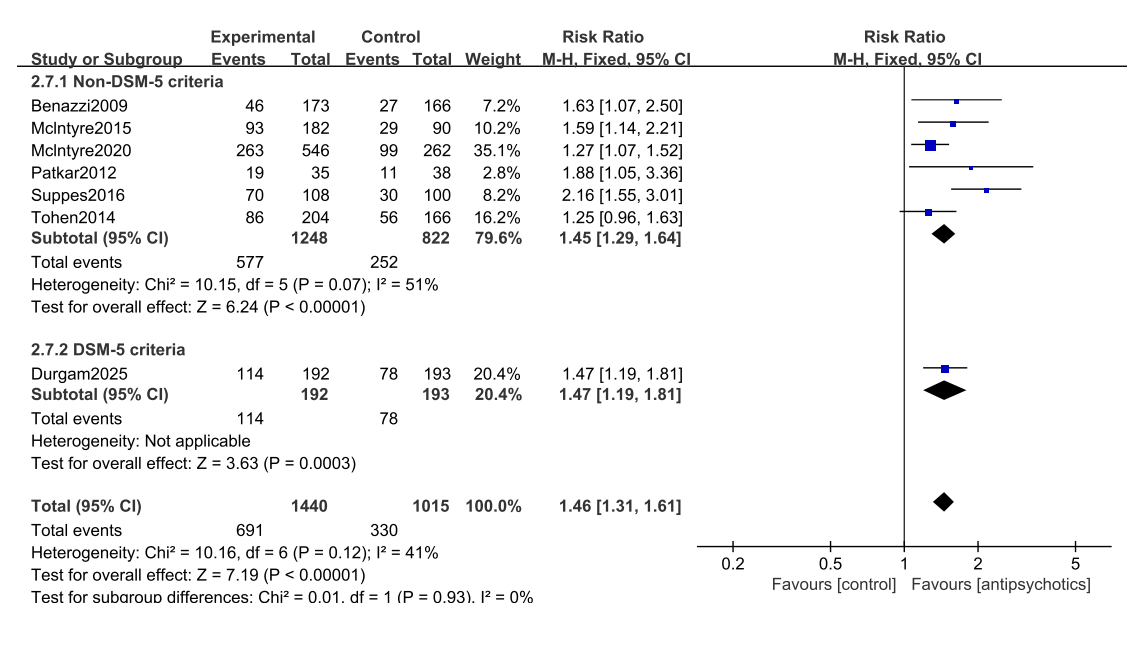


**Supplementary Figure 15** Forest plot for the clinical response rate of antipsychotics vs. placebo in subgroup analyses based on diagnostic criteria (non-DSM-5 vs. DSM-5).


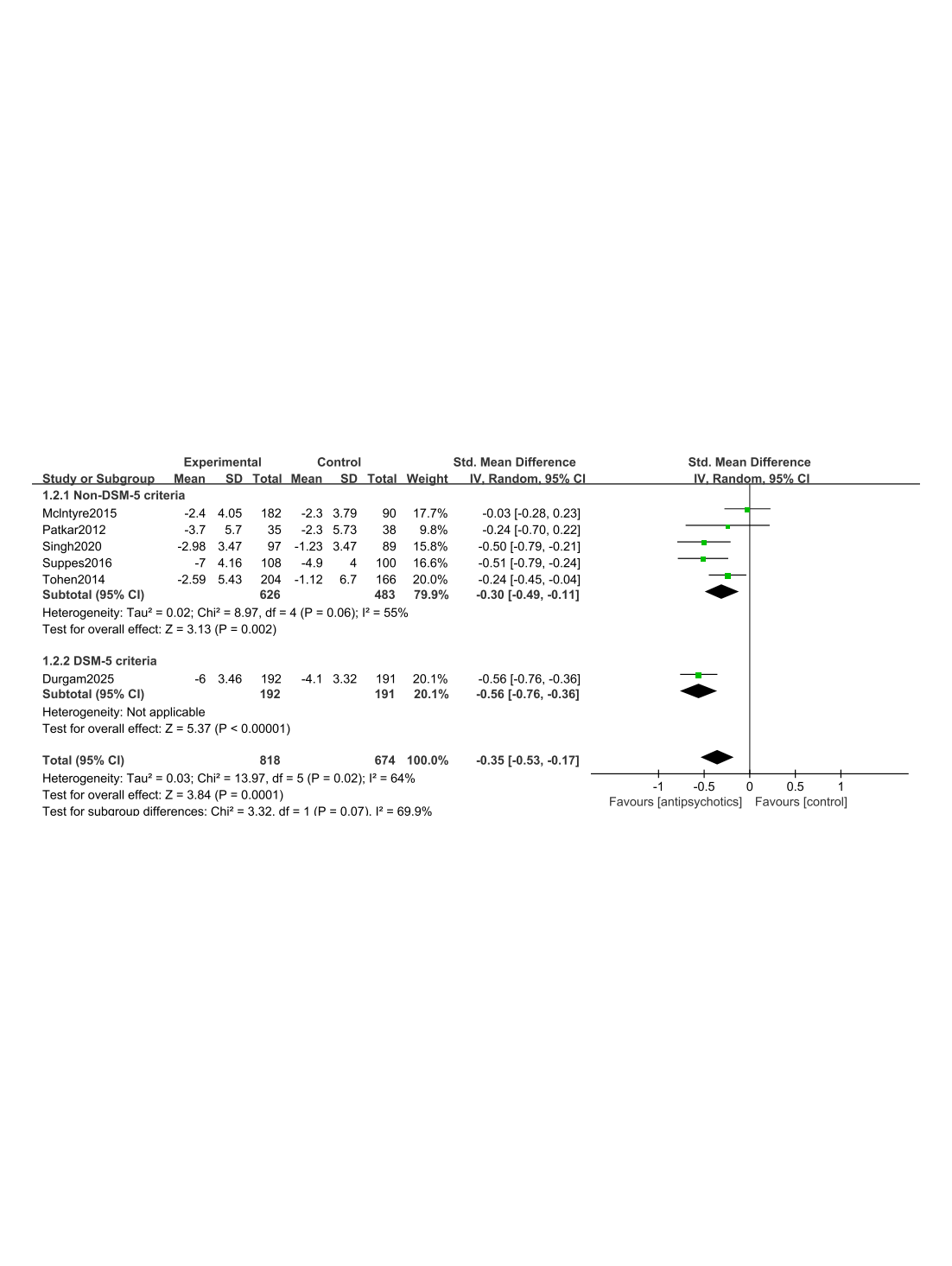


**Supplementary Figure 16** Forest plot for the changes in the YMRS score from baseline in patients with MDE-MFS treated with antipsychotics vs. placebo in subgroup analyses based on diagnostic criteria.


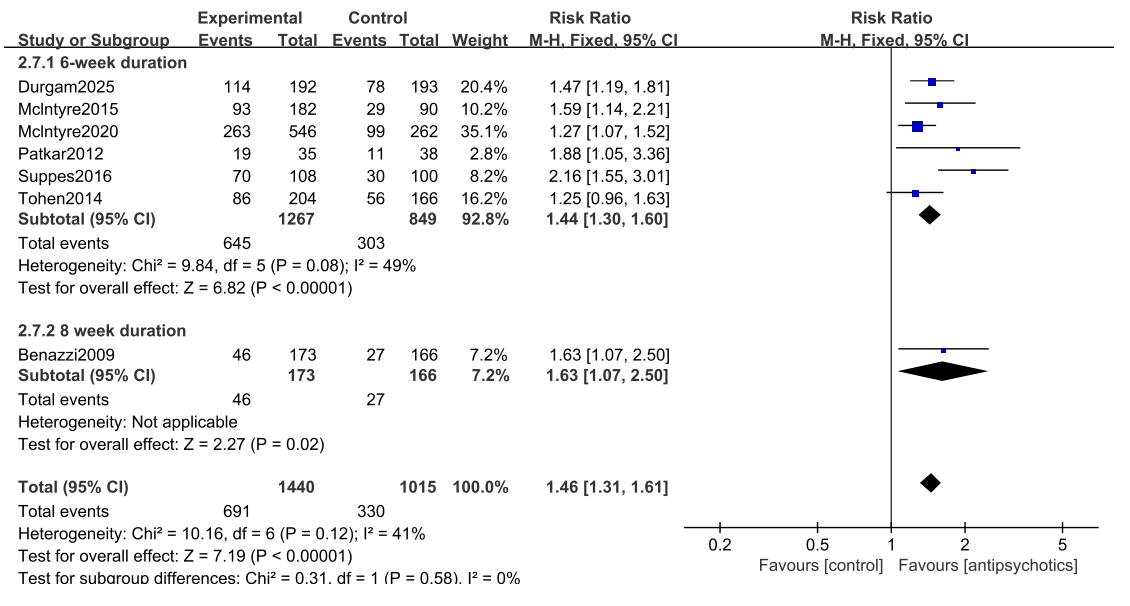


**Supplementary Figure 17** Forest plot for the clinical response rate of antipsychotics vs. placebo in subgroup analyses based on diagnostic criteria treatment duration (6-week vs. 8-week).
